# Ring-Opening Polymerization for the Goal of Chemically Recyclable Polymers

**DOI:** 10.1021/acs.macromol.2c01694

**Published:** 2023-02-06

**Authors:** Christopher M. Plummer, Le Li, Yongming Chen

**Affiliations:** † International Centre for Research on Innovative Biobased Materials (ICRI-BioM), Lodz University of Technology, Zeromskiego 116, 90-924 Lodz, Poland; ‡ Key Laboratory for Polymeric Composite and Functional Materials of Ministry of Education, Sun Yat-sen University, Guangzhou 510275, P. R. China; § School of Chemistry, Sun Yat-sen University, Guangzhou 510275, P. R. China; ∥ School of Materials Science and Engineering, Sun Yat-sen University, Guangzhou 510275, P. R. China

## Abstract

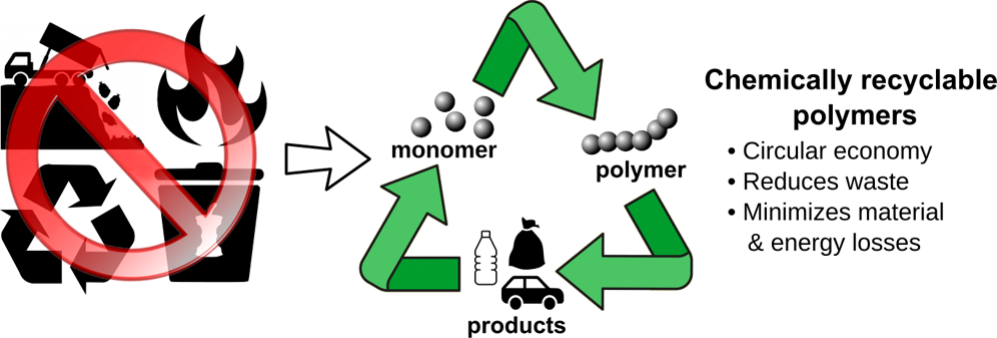

A crucial modern dilemma relates to the ecological crisis created by excess plastic waste production. An emerging technology for reducing plastic waste is the production of “*chemically recyclable*” polymers. These polymers can be efficiently synthesized through ring-opening polymerization (ROP/ROMP) and later recycled to pristine monomer by ring-closing depolymerization, in an efficient circular-type system. This Perspective aims to explore the chemistry involved in the preparation of these monomer/polymer systems, while also providing an overview of the challenges involved, including future directions.

## 1 Introduction

The low-cost and durability of plastics has resulted in an annual worldwide production exceeding 335 million tonnes, of which most is eventually discarded into landfills or directly into the environment. By 2050, this number is predicted to reach 1.12 billion tonnes annually.^1^ Plastics that float frequently end their lifespan in the ocean, with the ocean being the final destination for an estimated 5 million tonnes of plastic per year.^2^ In fact, single-use items constitute the major proportion of waste found in both marine and nonmarine environments.^2,3^ In response, the European Union (EU) has recently ruled a ban on single-use plastics, focusing on items including plastic bags, wrappers, cutlery, and straws.^4^ Due to the physical fragmentation of plastic wastes, microplastics are now also becoming pervasive throughout the environment. It is reported that microplastics are even contaminating human food items via integration into the food chain and contamination during production.^5,6^ As of now, the long-term consequences of microplastic waste on human health and the environment has yet to be established.

**Figure 1:**
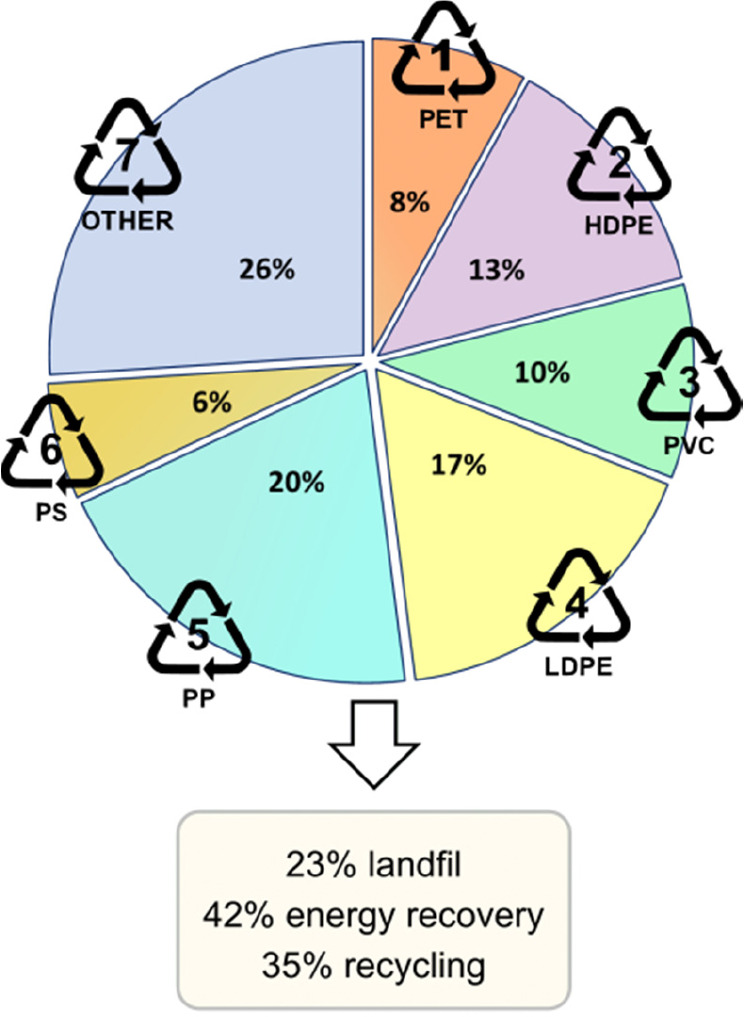
Demand and disposal method for plastics within the European Union in 2021/2020, respectively. Data source: Plastics–the Facts 2022, Plastics Europe.^10^

At present, six commodity plastics dominate the market: (1) polyethylene terephthalate (PET), (2) high-density polyethylene (HDPE), (3) polyvinyl chloride (PVC), (4) low-density polyethylene (LDPE), (5) polypropylene (PP), and (6) polystyrene (PS) (Figure 1). Although each of these plastics has a recycling code, this does not ensure that they are actually recyclable, with this differing from location to location. Five common approaches can be identified for addressing the production of waste plastic: landfilling, incinerating, recycling, biodegradable polymers, and chemically recyclable polymers. A classical method for plastic disposal involves burying the plastic in landfill; however, most plastics are essentially nondegradable, and any measurable degradation that does occur will leach chemicals into the environment. In addition, as plastics account for ca. 4–6% of total oil consumption, and as fossil fuels are a finite resource, it is unwise to squander such resources.^7^ The incineration of plastic waste to generate energy can recover a fractional portion of the embedded energy, although this process typically generates toxic waste and gas.^8,9^ Subsequently, landfill disposal and incineration both generate significant environmental pollution and recover only minimal value from the material.

Mechanical recycling can perhaps be considered the most recognizable sustainable response for controlling plastic waste. It involves first sorting and washing the postconsumer plastic waste before melt processing it to form new materials. Unfortunately, due to residual catalysts, moisture, and other contaminants, as well as thermal and shear-induced forces, each time a polymeric feedstock is recycled it is degraded by chain-scission, chain-branching, cross-linking, and oxidation. These phenomena negatively affect the physical properties of the resulting material, and thus a significant proportion of the recycled plastic is actually “downcycled”, or instead valorized by mixing it with virgin polymer.^9^ This limits the number of reprocessing cycles that a material can be subjected to, and therefore most polymers are inevitably landfilled or incinerated. As an example, within the European Union (EU) only 35% of collected polymer waste is recycled while 42% is incinerated for energy recovery, and 23% is sent directly to landfill.^10^

An environmentally conscious approach for reducing plastic waste is the production of biodegradable polymers. Such polymers are typically derived from biorenewable resources, and once discarded can be decomposed into CO_2_, H_2_O, CH_4_, and humic acid by enzymatic or hydrolytic activity, leading to an environmentally closed system.^11^ While materials such as poly(lactic acid) (PLA) and poly(glycolic acid) (PGA) have been advertised as sustainable and biodegradable solutions to the plastic waste crisis, their cost of production, material properties, and chemical durability are incomparable to present-day commodity polymers.^12^ Although being labeled as (bio)degradable, in reality such polyesters will often not readily degrade in the natural environment.^3,13,14^ Another drawback relates to their poor material properties, for example, low glass transition temperature (*T*_g_), which indicates they cannot be used for hot food or beverages. Moreover, the biodegradation of polymer waste can be considered inefficient as none of the feedstock material or energy value is recovered.^11,15^

**Figure 2:**
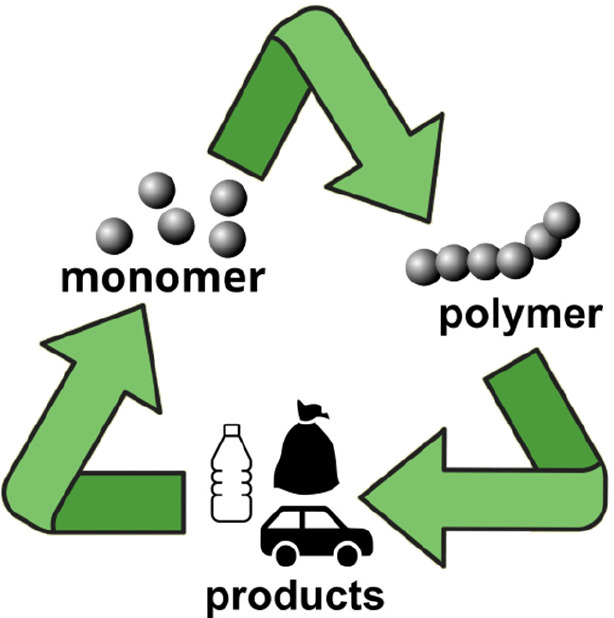
Lifecycle of a chemically recyclable polymer.

With the goal of zero landfilling of plastic waste and an authentic circular economy, new approaches to polymer chemistry must be devised. The fifth and most cutting-edge approach involves a recycling process in which postconsumer polymer waste is depolymerized under controlled conditions to yield monomer feedstock, which can then be purified and repolymerized. This emerging approach has been termed *chemical recycling* (Figure 2). As chemically recyclable polymers can be depolymerized back to pristine monomer feedstock, the recovered monomer can be used to produce polymer of virgin quality, therefore establishing a genuine closed-loop lifecycle. Moreover, as no energy is lost in the form of raw petrochemical feedstock or energy input into the system during the synthesis of the monomer, this mitigates other environmental effects.

For a chemically recyclable polymer to be considered commercially viable, it would need to be able to be depolymerized under mild conditions so that the process was energy efficient and would not cause concurrent decomposition. Conversely, the polymer would also need to be thermally stable during its service life. Moreover, to compete with modern commodity polymers, any new polymer would need to equal or exceed their material properties. Other challenging requirements include a low cost of synthesis and an efficient production at scale. The monomer design of chemically recyclable polymers therefore requires a judicious balancing of polymerizability, depolymerizability and performance, until a practical balance is achieved. As such, the development of monomers for next-generation polymers requires an authentic understanding of the relationship between molecular structure and the thermodynamics of (de)polymerization.

In general, polymerization is favored in enthalpy (Δ*H*) and disfavored in entropy (Δ*S*). The temperature at which the entropy loss will offset the enthalpy gain can be defined as the ceiling temperature (*T*_c_) of a polymerization system. Several factors such as monomer concentration, pressure, and state effect this value. Polymerization is favored when the temperature is below the *T*_c_ value, and conversely, depolymerization is favored above the *T*_c_ value. Equally, when a polymerization system is held at the *T*_c_ the rate of polymerization and depolymerization are equivalent. An important factor that needs to be addressed for a chemically recyclable polymer system is the balance between depolymerizability and thermal stability. If a material has a low *T*_c_ value, it may depolymerize at a catastrophic time during its working lifespan. Polymers such as polyolefins have high *T*_c_ values and their depolymerization is therefore both exorbitant from an energy standpoint, as well as prone to degradation. Conversely, low *T*_c_ polymers such as poly(α-methylstyrene) lack the required thermal stability for commercial use.^16^ Polymers derived from moderately strained heterocyclic monomers can be identified as viable candidates for chemical recycling because of their moderate *T*_c_ values of <250 °C.^17^

Ring-opening polymerization (ROP) is a chain-growth polymerization technique which encompasses many propagation mechanisms including cationic, anionic, radical, and coordination–insertion. Ring-opening metathesis polymerization (ROMP) involves the transition metal-catalyzed metathesis polymerization of cyclic olefins. The relative success of ring-opening polymerizations (ROP/ROMP) in the field of chemically recyclable polymers can be specifically attributed to the milder enthalpy change of cyclic monomers during polymerization, which therefore means a lower *T*_c_ value in comparison to vinyl polymers.^18^ The principal strategy for reducing the entropic penalty of a ring-opening polymerization is to perform the polymerization at a temperature below the *T*_c_ value of a polymerization system. A second strategy involves carefully choosing polymerization conditions (concentration, temperature, solvent) that force the formed polymer to crystallize out of solution, thereby continuously shifting the equilibrium toward polymerization.^8,19^

**Figure 3:**
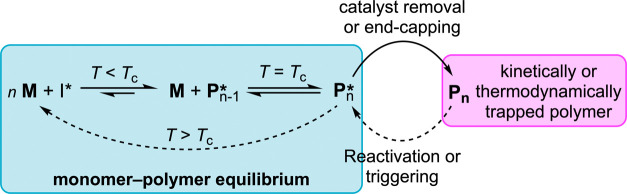
Monomer–polymer equilibriums, and the stabilization of chemically recyclable polymers above their ceiling temperature (*T*_c_).

In concept, a polymer system could be designed that could be simply depolymerized by holding it above its *T*_c_ value without a catalyst. However, the application of catalysts offers an opportunity to perform (de)polymerization in an efficient manner at a lower energy barrier, thereby creating many advantages. It is also possible to create a monomer/polymer system that can only undergo (de)polymerization in the presence of a catalyst capable of significantly lowering the energy barriers. In such a system, once the active polymerization is quenched and the catalyst deactivated or removed, the polymer thereafter exists in a “kinetic trap” and is effectively removed from the monomer–polymer equilibria (Figure 3). This can provide a polymer with stability during its working lifecycle, even at temperatures above its *T*_c_ value. Reactivation from the trapped state to the equilibrium state may be achievable by the addition of high amounts of energy into the system to overcome the kinetic barrier, or instead, by the addition of a catalyst that can promote a low-energy pathway for its depolymerization.^19^ An additional strategy for the production of “stable” chemically recyclable polymers is chain-end-capping, with polymers containing this property often termed self-immolative polymers (SIPs).^20,21^ Chain-end-capped polymers depolymerize head-to-tail upon cleavage of their preinstalled end-cap groups, and in this context such polymers can be considered “thermodynamically trapped” (Figure 3). Such chain-end-capping requires a stoichiometric amount of capping reagent and a living polymerization process to be effective. In addition, reactive chemicals (or an external stimuli) are required to cleave the end-cap and trigger depolymerization, and therefore this approach can be considered a relatively less attractive approach for the preparation of commodity plastics.

The synthesis of chemically recyclable polymers is a broad research area with several recent developments. Accordingly, there have been multiple excellent review articles published that overlap with the topics discussed herein.^11,15,19,22,23^ This Perspective chooses to focus on chemically recyclable polymers produced by the application of ring-opening polymerization technologies. It is divided into two sections: (1) monomers that (de)polymerize by ROP and (2) monomers that (de)polymerize by ROMP, which are then further divided by monomer type. The article principally focuses on monomers that provide linear (noncyclic) polymers which depolymerize to return monomer in excellent yield without major side product, and additionally do not require end-capping for polymer stability. Although not all the monomers described within this article can be considered viable candidates for chemically recyclable plastics, they are included so that a thorough overview is provided regarding the various properties that can be obtained, thereby assisting the targeted development of next-generation monomers and polymers.

## 2 Ring-Opening Polymerization (ROP)

### 2.1 Cyclic Ethers

Polytetrahydrofuran (**poly1**), also known as polytetramethylene ether glycol, is a waxy white solid with a low melting transition temperature (*T*_m_) of 30–50 °C, dependent upon the molecular weight, for which values between 250 and 3000 kg mol^–1^ are typical. Its primary use is the synthesis of multicomponent polymers such as polyesters and polyurethanes by its reaction with diacids and diisocyanates, respectively. **Poly1** is readily synthesized by the ROP of tetrahydrofuran (**1**) in the presence of suitable catalysts such as Brønsted or Lewis acids. Of note is that tetrahydrofuran (**1**) can be prepared by the hydrogenation of furan, which can in turn be obtained from furfural which is a renewable resource prepared from agricultural waste. Within the patent literature there exists multiple vaguely described protocols for the depolymerization of **poly1** using catalysts such as rare-earth catalysts, kaolin, zeolite, aluminum silicates, and sulfuric acid.^24−28^

**Figure 4:**
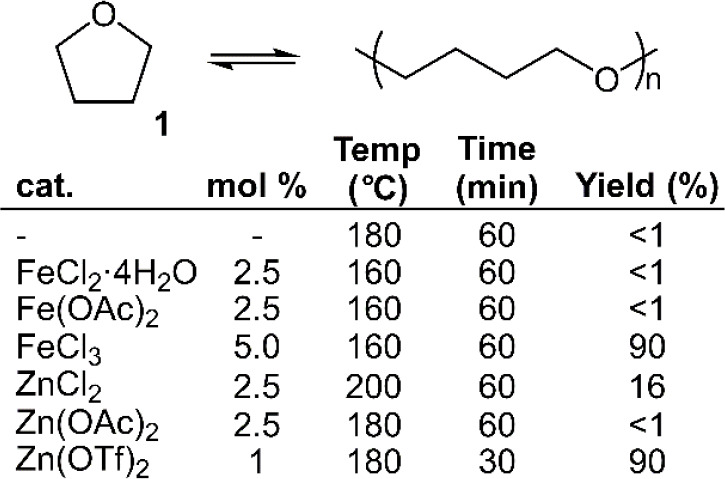
Depolymerization of tetrahydrofuran (**1**) using various metal salts.

Using these conditions as a lead, Enthaler and Trautner explored the depolymerization of **poly1** using simple iron salts.^29^ It was reported that depolymerization could be performed by distillation at 160 °C using FeCl_3_ as a catalyst, returning **1** in 90% yield (Figure 4). Moreover, it was reported that the catalyst was reusable for multiple cycles with little loss of catalytic activity. Multiple alternative iron-containing salts were screened for their catalytic activity, but in all cases no significant monomer formation was observed. Enthaler later explored the depolymerization of **poly1** using Zn(OTf)_2_, reporting that depolymerization at 180 °C could furnish 90% recovered monomer after only 30 min.^30^ A variety of zinc salts were screened and reported to be nonuseful, with the exception of ZnCl_2_ which provided a low yield of 16% at 200 °C. Song et al. later examined depolymerization using various heteropolyacids, reporting that the use of phosphotungstic acid (10 wt %) at 130 °C for 15 min could provide **1** in yields greater than 95%, and was also reusable over many cycles with only a minor loss in catalyst activity.^31^

### 2.2 Cyclic Esters

**Figure 5:**
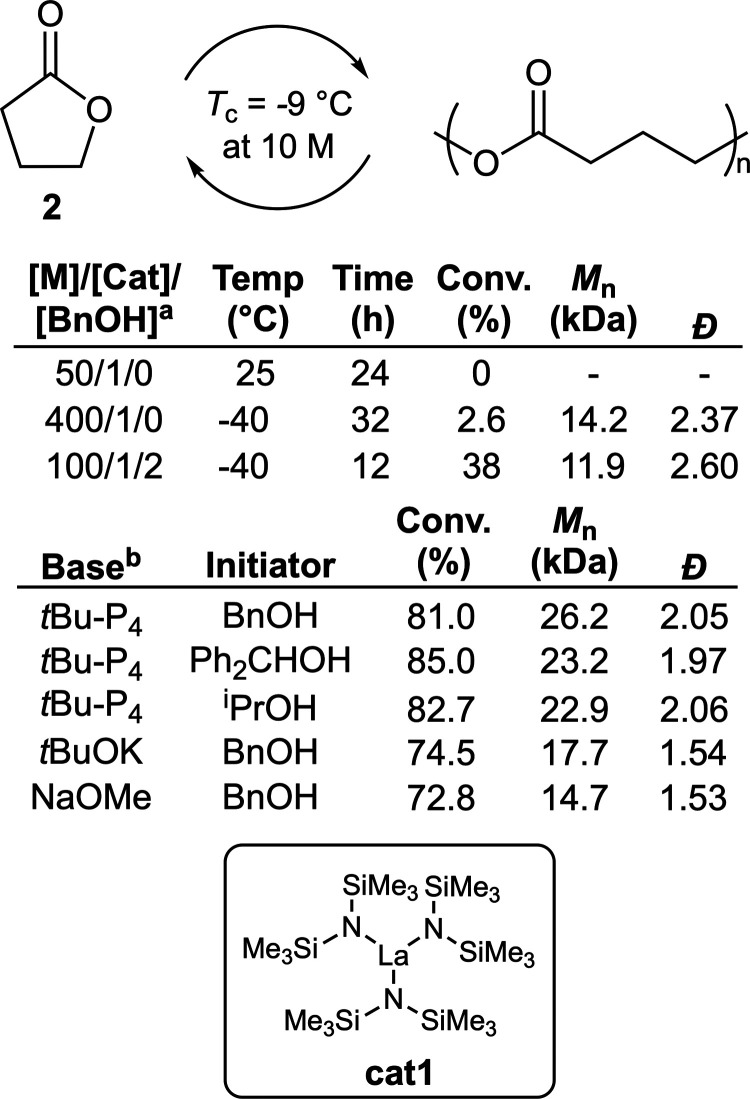
Polymerization conditions: ^a^solvent = toluene; ^b^[M]/[Base]/[Initiator] = 100/1/1, respectively, temperature = −40 °C, solvent = THF, reaction time = 4 h.

γ-Butyrolactone (**2**) has been ranked among the top biomass-derived compounds to replace petroleum-derived chemicals. However, historically it has been considered nonpolymerizable due to low ring-strain. In reality, the synthesis of **poly2** is possible under ultrahigh pressure (20,000 atm at 160 °C); however, this process can only produce oligomers with a *M*_w_ of <5 kg mol^–1^. The reluctance of **2** to polymerize arises from the unfavorable thermodynamics of ROP originating from the low ring-strain energy which brings about a too small negative change in enthalpy to offset the large entropic change due to the polymerization process. Nevertheless, in 2016 Chen and co-workers reported that **2** can polymerize under ambient pressures with a suitable catalyst, producing materials with *M*_n_ values of ≤30 kg mol^–1^ (Figure 5).^32^ This was achieved by a dual strategy of (a) performing the polymerization below the *T*_c_ value for a given monomer concentration, and (b) performing the polymerization under conditions where the polymer precipitates, forcing the equilibrium to shift toward polymerization. Guided by these two strategies, the ROP of **2** was first performed using 2.0 mol % La[N(SiMe_3_)_2_]_3_ (**cat1**) as a catalyst at 10 M concentration in toluene at −40 °C, these conditions allowing the formed polymer to precipitate as the polymerization proceeded, providing a polymer sample with *M*_n_ = 12.1 kg mol^–1^ (*Đ* = 1.99). Unfortunately, the monomer conversion of **2** was limited to ca. 3% regardless of the polymerization conditions. Additionally, analysis by MALDI-TOF revealed that the polymer was actually cyclic, ascribed to intramolecular backbiting.

The addition of BnOH as an initiator in a [M]/[Cat]/[BnOH] ratio of 100/1/2, respectively, was reported to be able to raise the total monomer conversion to 38% (Figure 5). Further exploration revealed that the polymer macrostructure was dependent on the [La]/[BnOH] ratio; with a ratio of 1/3 giving linear polymer with only traces of cyclic impurity. It was reported that TGA analysis of **poly2** could conveniently assess the polymer topology (i.e., linear versus cyclic) via their distinctive temperature of degradation (*T*_d_) values. It was also reported that the use of Ph_2_CHCH_2_OH as an initiator could provide samples without a detectable cyclic component. Thermal depolymerizations of **poly2** were reported possible at 220 °C, giving quantitative conversion. It was also reported that depolymerization could be performed at 25 °C in the presence of organic or metal catalysts. A follow-up publication exploring the use of superbase *t*Bu-P_4_ revealed that this system could provide **poly2** with monomer conversions up to 90% and *M*_n_ values as high as 26.7 kg mol^–1^ (Figure 5).^33^

**Figure 6:**
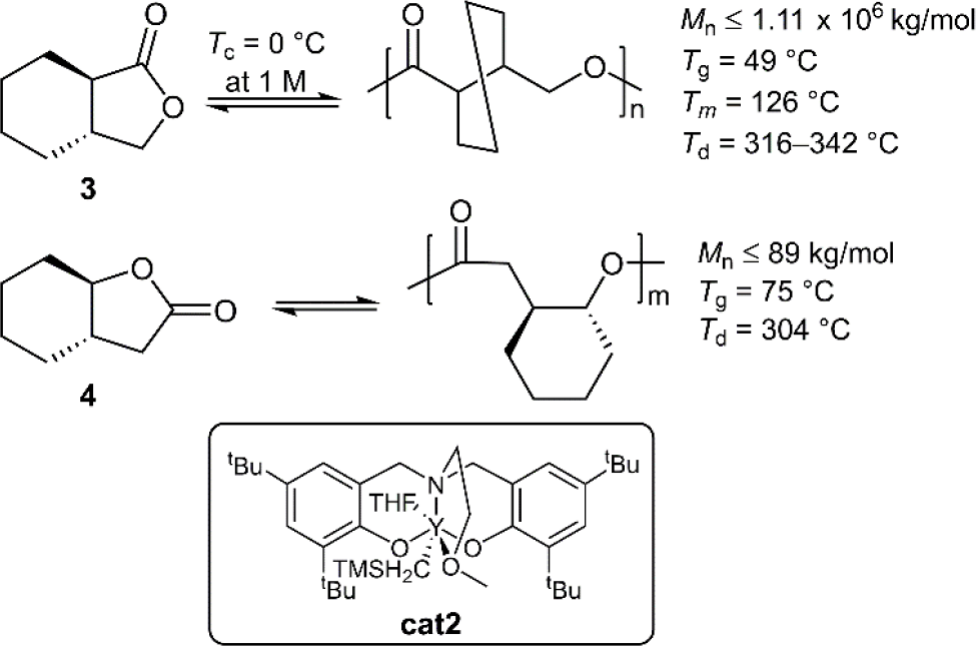
Polymerization of cyclohexane fused γ-butyrolactone monomers.

Although the synthesis of **poly2** was a major milestone, commercially its polymerization can be considered unattractive due to demanding conditions (−40 °C) and limited thermal stability of the polymer (*T*_m_ = ∼ 63 °C and *T*_d_ = 202 °C). It was theorized that the thermodynamic properties could be tuned by substitution of the γ-butyrolactone ring. In this way, *trans*-3,4-cyclohexane fused **3** was synthesized and examined for its (de)polymerizability (Figure 6).^34^ When **3** was subjected to ROP with 0.1 mol % **cat1** and an alcohol initiator, it was reported to provide monomer conversions of up to 80% at 25 °C. Moreover, it was reported that yttrium catalyst **cat2** could provide linear **poly3** with *M*_n_ values as high as 11.1 × 10^6^ g/mol (*Đ* = 1.09), the value being readily controllable by the monomer/catalyst ratio. Unusually, it was reported that some catalysts caused irreversible isomerization to the nonpolymerizable *cis*-isomer.

Heating **poly3** at ≥300 °C for 1 h was reported to give quantitative depolymerization to the monomer. Depolymerization was also reported to be quantitative at 120 °C when a catalytic metal salt (i.e., ZnCl_2_) was applied. The (de)polymerization cycle was described to be successful on a multigram scale over three consecutive cycles, providing *ca*. 96% monomer recovery. To address the fact that racemic **poly3** is an atactic amorphous solid with poor material properties, isotactic **poly**(***R***,***R***-**3**) and **poly**(***S***,***S***-**3**) were prepared from enantiopure monomer feedstock. The two isotactic polymers were subsequently applied in an equimolar blend to give stereocomplexed material ***sc***-**poly3**. This material showed a persistent higher *T*_m_ value of 188 °C in comparison to isotactic **poly3** (*T*_m_ = 126 °C) which only showed melting transitions in the first heating cycle, indicating a slow crystallization rate. To simplify the synthesis of ***sc***-**poly3**, a catalytic system capable of performing the stereoeselective ROP of ***rac***-**3** was achieved using yttrium catalysts.^35^ This system was able to provide isotactic stereoblock polymers which could be used to prepare ***sc***-**poly3** with a *T*_m_ value of 171 °C, this being able to be depolymerized to ***rac***-**3** thereby establishing proper circular recyclability.

In addition, the ROP of an alternative *trans*-cyclohexane fused γ-butyrolactone, monomer **4**, was reported to provide polymers with *M*_n_ values ≤ 89 kg mol^–1^, high thermal stability, and chemical recyclability (Figure 6).^36^ Comparisons of **poly4** with its constitutional isomer **poly3** revealed that **poly4** exhibits a higher *T*_g_ value (75 °C versus 49 °C, respectively) but a marginally lower *T*_d_ value. Unfortunately, the depolymerization of **poly4** was reported to be problematic owing to temperature-induced isomerization to give the nonpolymerizable *cis*-isomer (i.e., ***cis***-**4**). To suppress isomerization, the depolymerization was performed in the presence of **cat1** at 120 °C which provided quantitative depolymerization without isomerization.

**Figure 7:**
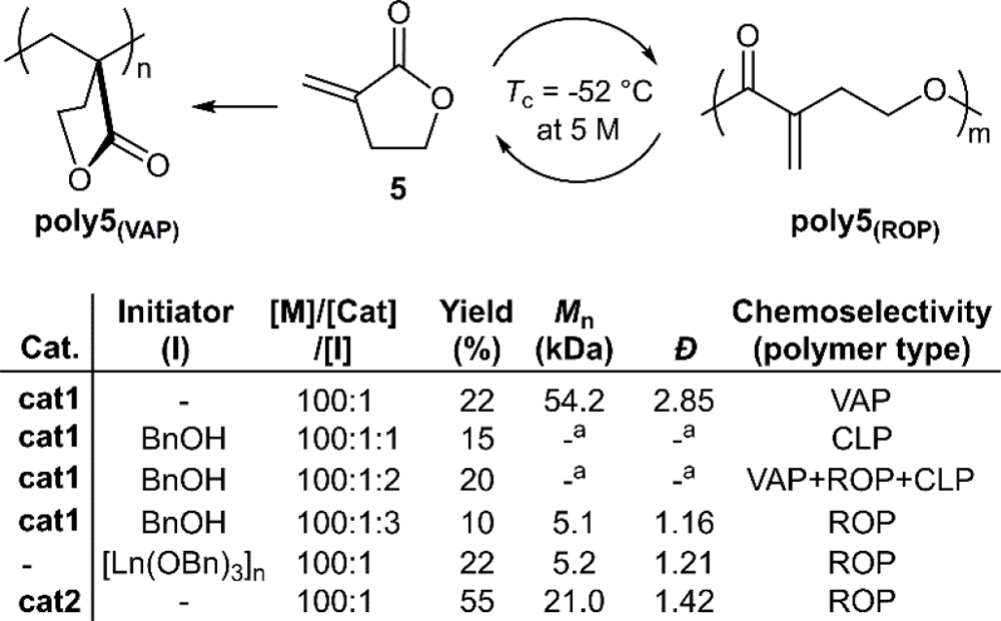
Polymerization conditions: 5 M initial monomer concentration in THF at −60 °C. ^a^Not determined due to poor solubility.

Tulipalin A (**5**) is a naturally occurring monomer that is reported to readily undergo vinyl addition polymerization (VAP) to give **poly5**_**(VAP)**_ (Figure 7). It was anticipated that disabling the VAP mechanism to favor ROP would be possible, thereby enabling the synthesis of **poly5**_**(ROP)**_.^37^ Lanthanum complex **cat1** was examined for polymerization but was reported to exclusively provide **poly5**_**(VAP)**_. It was later theorized that the addition of an alcohol species could improve its performance by an in situ alcoholysis to generate a La-alkoxide catalyst. The addition of BnOH at an equimolar Ln/BnOH ratio was reported to give an insoluble cross-linked polymer (labeled CLP), while a ratio of 1:2, respectively, provided a sample likely formed by all three polymerization mechanisms operating simultaneously (VAP, ROP and CLP). Unexpectedly, a 1:3 ratio, respectively, finally provided a sample of the elusive **poly5**_**(ROP)**_ (*M*_n_ = 5.1 kg mol^–1^, *Đ* = 1.16), albeit with low monomer conversion (10%). It was reported that polymerization with an isolated sample of [Ln(OBn)_3_]_n_ also led to the formation of **poly5**_**(ROP)**_, proving the catalyst hypothesis correct. Yttrium complex **cat2** was reported to be a superior polymerization catalyst, providing **poly5**_**(ROP)**_ on a multigram scale with *M*_n_ values of ≤21 kg mol^–1^ (*Đ* = 1.42), with improved monomer conversions up to 55%. **Poly5**_**(ROP)**_ was examined by TGA and reported to have a *T*_d_ of 293 °C, displaying two degradation steps which were attributed to initial cross-linking before degradation. It was described that **poly5**_**(ROP)**_ could be quantitatively depolymerized at 100–130 °C for 1 h, or 60 °C for 24 h, by heating in a 0.2 M DMSO solution in the presence of **cat1** (1 mol %) with 3.5 mM of H_2_O to inhibit polymerization. Moreover, it was reported that simple metal halides (i.e., LaCl_3_), which are incapable of reinitiating VAP polymerization, could also be applied for depolymerization.

**Figure 8:**
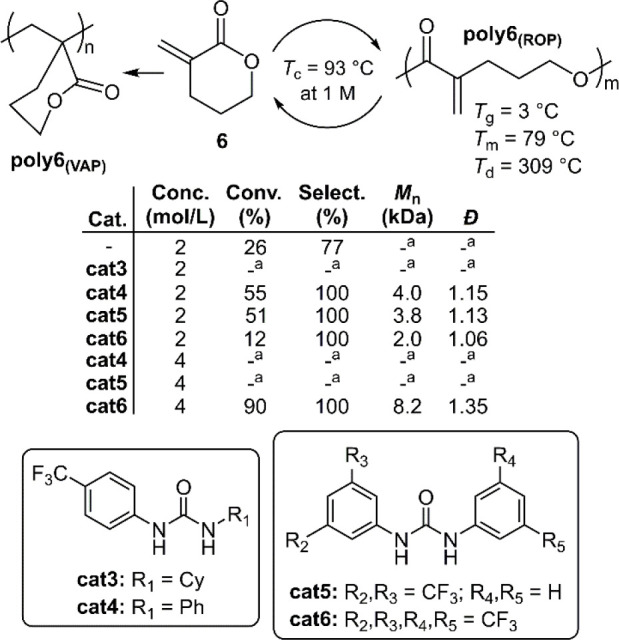
Polymerization conditions: performed using KOMe in THF at 25 °C with a [M]/[Base]/[Cat] ratio of 100/1/3, respectively, for 10 min; ^a^ not determined due to cross-linking.

Cyclohexane analogue **6** was also reported polymerizable using KOMe, achieving a monomer conversion of 26% and producing a mixture of vinyl addition and ROP product (**poly6**_**(VAP)**_ and **poly6**_**(ROP)**_, respectively) (Figure 8).^38^ When catalyst **cat3** was included in a [M]/[Base]/[Cat] ratio of 100/1/3, respectively, the polymerization rate improved but cross-linking occurred, presumed to be due to the action of both VAP and ROP pathways. The exploration of alternative urea catalysts (**cat5**/**cat6**) resulted in quantitative ROP at the expense of monomer conversion. Polymerizations were then performed at a higher concentration ([M]_0_ = 4 M) using **cat4** and **cat5**, however this system was reported to again lead to cross-linking. The exclusive ROP of **6** was finally achieved using **cat6**, providing **poly6**_**(ROP)**_ with an *M*_n_ = 8.2 kg mol^–1^ (*Đ* = 1.35). The *T*_c_ of **poly6**_**(ROP)**_ was reported to be 93 °C at 1 M, this value being considerably higher than its cyclopentane analogue **poly5** (−126 °C at 1 M). In combination with **cat6**, a variety of organobases were examined for the depolymerization of **poly6**_**(ROP)**_. It was reported that near-quantitative (96%) depolymerization could be achieved using **cat6** and *t*Bu-P_2_ in DMF at 80 °C for 24 h. Bulk depolymerization was also performed by heating **poly6**_**(ROP)**_ at 130 °C under reduced pressure in the presence of 0.5 wt % Sn(Oct)_2_, providing near-quantitative monomer recovery within 2 h.

**Figure 9:**
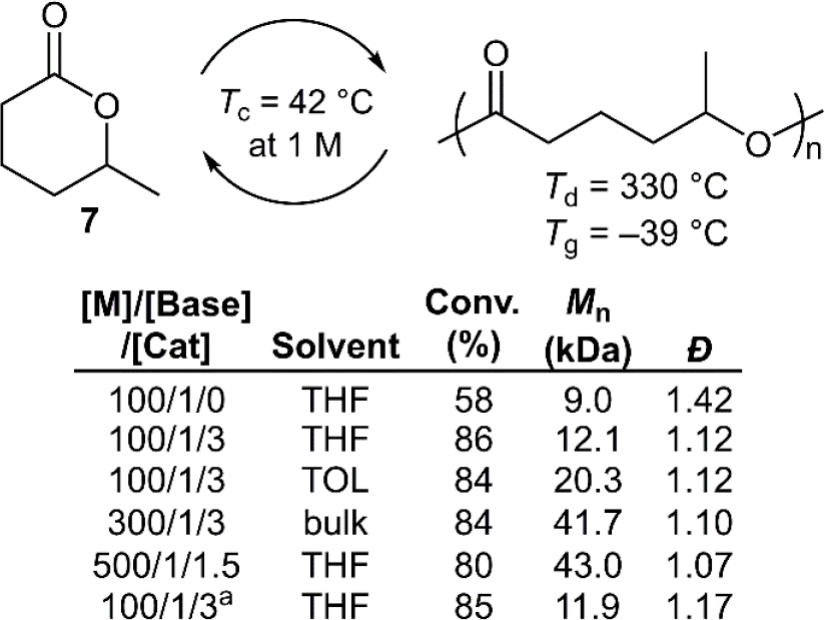
Polymerization conditions: performed using KOMe at 25 °C; ^a^using recovered monomer from depolymerization.

In 2022, Li et al. reported that cyclohexane analogue δ-caprolactone (**7**) could be readily (de)polymerized (Figure 9).^39^ ROP catalyzed by KOMe alone achieved a low monomer conversion of 58% and a relatively broad dispersity (*Đ* = 1.42). With the addition of urea cocatalysts, in particular **cat3**, it was reported that both the polymerization rate and monomer conversion could be improved significantly, giving **poly7** with *M*_n_ values of ≤41.7 kg mol^–1^ (*Đ* = 1.10). Polymers with higher *M*_n_ values could be prepared by a solvent exchange of THF to toluene, predicted to be due to improved catalyst solubility. It was reported that the depolymerization of **poly7** gave 94% monomer recovery within 1 min using organophosphazene superbase CTPB, but that no depolymerization occurred when using weaker bases. Unusually, it was even reported that **poly7** remained stable in the presence of 1,8-diazabicyclo[5.4.0]undec-7-ene (DBU) and triethylamine (TEA) for up to 5 days. Bulk depolymerization was examined in the presence of 0.5 wt % Sn(Oct)_2_ at 130 °C, providing quantitative monomer recovery within 2 h by vacuum distillation.

**Figure 10:**
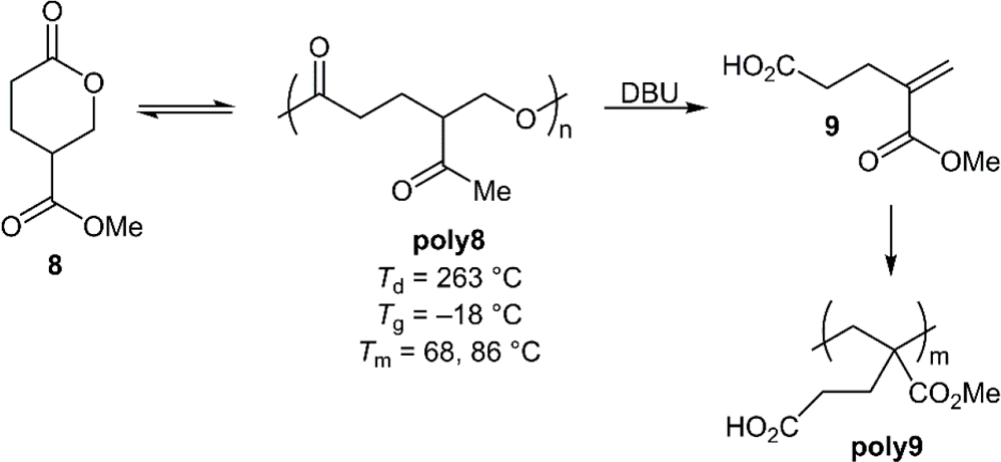
Distinct depolymerization pathways of **poly8**.

In 2018, a polymer (**poly8**) was described that was reported capable of being chemically recycled by two distinct depolymerization pathways (Figure 10).^40^ Its precursor, monomer **8** is readily synthesizable in two steps from malic acid, a renewable feedstock. The ROP of **8** was performed using 1,4-benzenedimethanol and DPP at 25 °C, giving polymer with *M*_n_ values of ≤54.3 kg mol^–1^ (*Đ* = 1.2). **Poly8** was reported to be semicrystalline, having a low *T*_g_ of −18 °C and two *T*_m_ values of 68 and 86 °C, with this being considered unusual as **8** is a racemic mixture and thus **poly8** is likely atactic. It was reported that **poly8** was chemically recyclable under vacuum using Sn(Oct)_2_ as a catalyst at 150 °C, providing 87% monomer recovery. Moreover, by the application of DBU, **poly28** was also reported to be depolymerizable to monomer **9** in 88% monomer yield. Recovered **9** could be repolymerized to provide a unique polymethacrylate derivative (**poly9**). This system therefore constitutes a rare example of a polymer having multiple distinct chemical recycling pathways.

**Figure 11:**
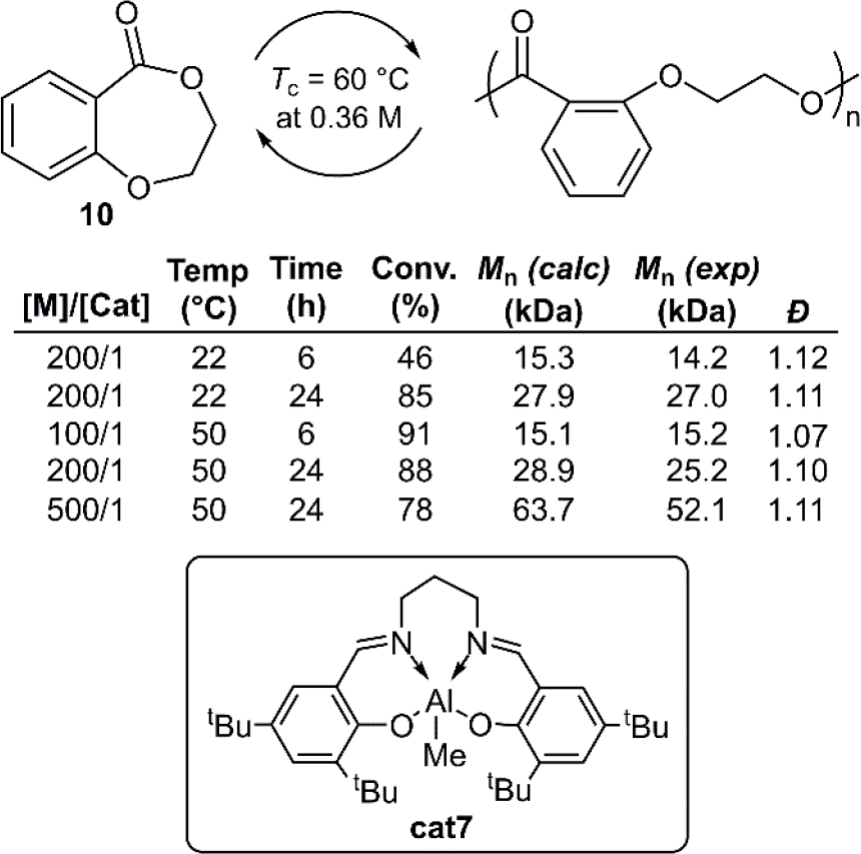
Polymerization of benzodioxepinone 10 conducted in toluene using MeAl[salen] complex cat7.

Polymerizations of monomer **10** using aluminum salen complex **cat7** were reported to be efficient and well-controlled, providing polymers with *M*_n_ values of ≤52.1 kg mol^–1^ (*Đ* = 1.11) (Figure 11).^41^ Polymerization under dilute conditions resulted in low monomer conversion, and therefore high initial monomer concentrations (4–5 M) were applied. However, it was later discovered that polymerizations catalyzed by triazabicyclodecene (TBD) could successfully catalyze polymerization at low concentration. For example, polymerizations with an [M]_0_ value = 2.4 M reached 87% conversion within 30 min, although further decreases in monomer concentration resulted in lower conversion. It was reported that the addition of BnOH as a co-initiator could afford copolymers with *M*_n_ values of ≤78.6 kg mol^–1^ (*Đ* = 1.22).^42^

**Poly10** was reported to have a *T*_g_ value of 31 °C, but only had a detectable *T*_m_ value during the first heating cycle, thereby indicating slow crystallization.^42^**Poly10** was reported to have a degradation temperature of 146 °C which can be considered low even for a polyester (e.g., polylactic acid *T*_d_ = 290.5 °C) thereby limiting any commercial value. To examine **poly10** depolymerization, a polymerization was performed using **cat7** with an [M]_0_ value of 4.1 M for 6h at 60 °C to give a monomer conversion of 82%. The addition of toluene to this system to give an apparent [M]_0_ of 0.2 M resulted in almost quantitative depolymerization. Subsequent concentration of the system to give an apparent [M]_0_ of 4.1 M resulted in 84% monomer conversion to **poly10** after 6h, thereby demonstrating the ability of **10** to readily undergo circular (de)polymerization.

**Figure 12:**
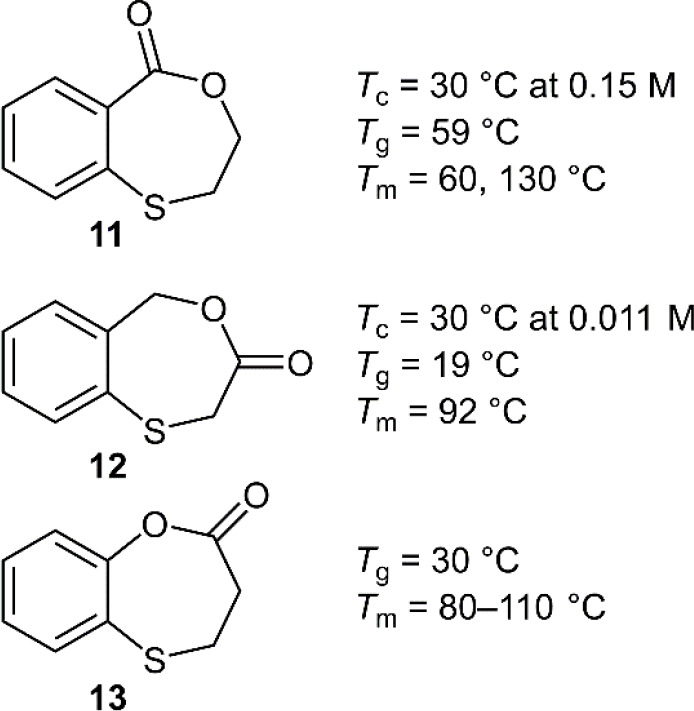
Thio-containing benzodioxepinone analogues. Note: *T*_g_ values were measured on the second heating cycle; *T*_m_ values were measured after annealing at a temperature above the *T*_g_ value for a week, and then allowing the sample to cool at 25 °C for multiple days.

To improve the material properties of this series, Li et al. examined three isomers of **10** incorporating sulfur atoms (Figure 12).^43^ Applying TBD as a catalyst and BnOH as an initiator at [M]_0_ = 2 M at 30 °C gave monomer conversions as high as 92%. For example, when the polymerization of **11** was performed with a [M]/[BnOH]/[TBD] ratio of 100/1/1, respectively, this provided **poly11** with 72% monomer conversion and a *M*_n_ value of 18.2 kg mol^–1^ (*Đ* = 1.09). When applying diphenyl phosphate (DPP) as a catalyst and BnOH as an initiator, *M*_n_ values of ≤17.3 kg mol^–1^ (*Đ* = 1.07) could be obtained, while the use of 1,4-benzenedimethanol (BDM) gave *M*_n_ values as high as 67.4 kg mol^–1^ (*Đ* = 1.11), but which were noted to contain a shoulder-peak in the SEC trace. Multiple catalysts were examined for the polymerization of **12** but only DPP could offer controlled polymerization. The *T*_c_ of **12** was determined to be 0.011 M at 30 °C, this low value implying that it can be readily converted to polymer at 30 °C, but conversely, will be difficult to depolymerize. Monomer **13** was reported to be polymerizable by multiple catalysts (excluding DPP) but was also reported to include significant quantities of cyclic oligomer. Moreover, the polymerization of **13** was noted to be difficult to control, ascribed to trans-esterification reactions occurring during ROP.

The thermal transitions of **poly11**–**poly13** were then examined by DSC. It was noted that **poly11**, which was obtained directly by precipitation, had no *T*_g_ value but did have a *T*_m_ value of 130–150 °C. During the second heating cycle a *T*_g_ value of 59 °C was measurable, but the *T*_m_ was absent. Interestingly, annealing this polymer above its *T*_g_ value for 1 week and then leaving it at 25 °C for multiple days provided a material that contained two distinct *T*_m_ values of 60 and 130 °C, indicating the coexistence of different crystal forms that poorly develop from the melt. Similar phenomena were reported for all three polymers, and therefore this monomer series can be considered semicrystalline materials with slow melt crystallization rates. The depolymerization of **poly11** in solution was reported to generate a mixture of both monomer and oligomer, the proportions dependent upon the conditions, while the depolymerization of **poly12** provided almost exclusively oligomer. Conversely, the depolymerization of **poly13** using TBD at 25 °C readily provided monomer. Due to the poor depolymerization performance of **poly11** and **poly12**, bulk depolymerizations were examined under vacuum at 200 °C using Sn(Oct)_2_ as a catalyst, with **poly11** providing 78% monomer recovery. It was reported that the yield could be further increased to 93% by the addition of poly(ethylene oxide) (PEO), with both the PEO and the catalyst remaining active for multiple depolymerization cycles. Under identical conditions, the depolymerization of **poly12** was also successful, but **poly13** was reported to be less thermally stable, giving low monomer recovery.

**Figure 13:**
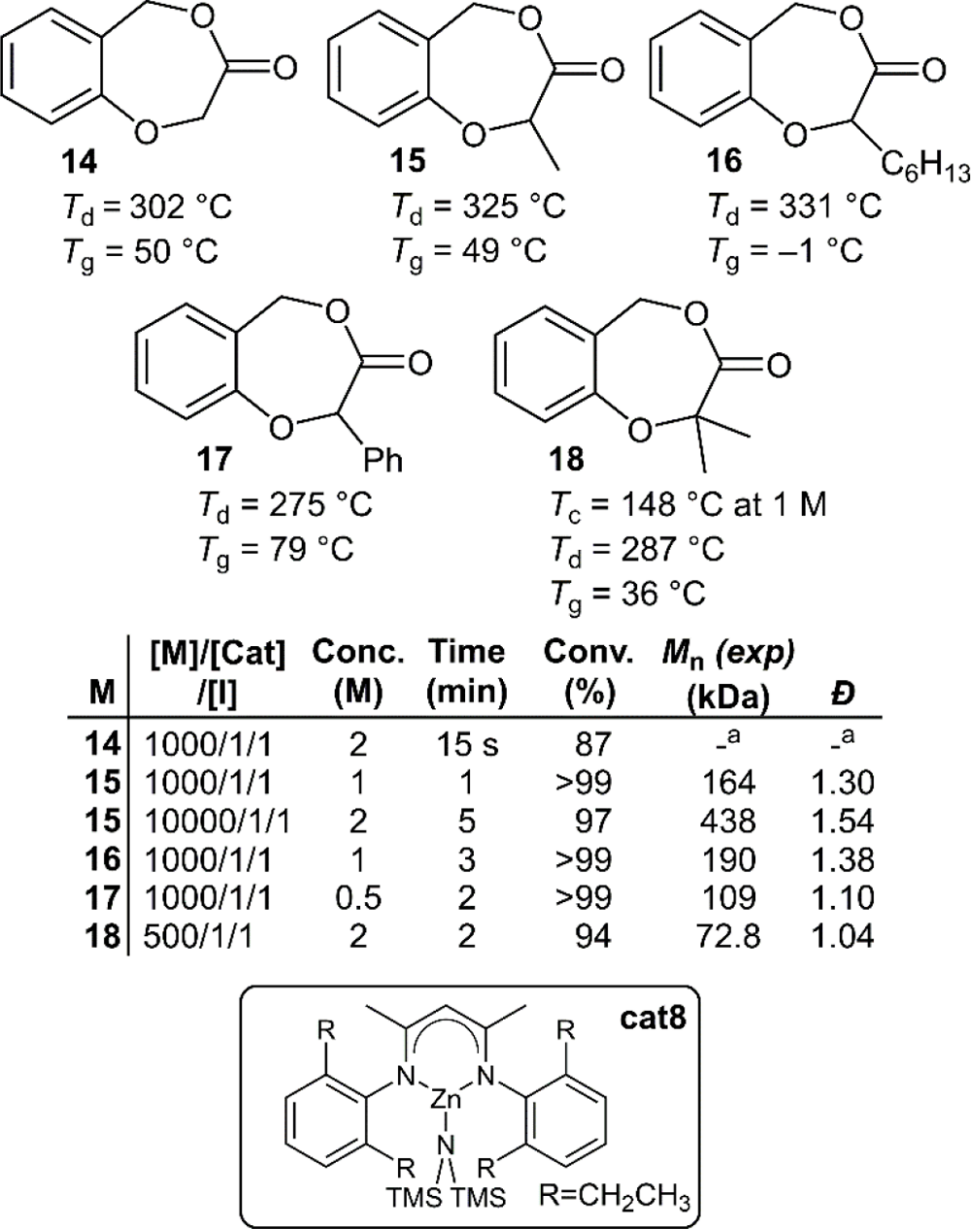
Polymerization conditions: performed in THF at 25 °C using cat2 and *p*-tolylmethanol as an initiator (I); ^a^not determined due to poor solubility.

It was speculated that repositioning the ester moiety of **10** to disable conjugation with the aromatic ring could enhance ROP activity, and consequently five additional benzodioxepinone analogues (**14**–**18**) were examined (Figure 13).^44^ Their ROP was reported to be efficient with **cat2** using *p*-tolylmethanol as an initiator at 25 °C, giving polymers with *M*_n_ values of ≥438 kg mol^–1^ (*Đ* = 1.54). Unusually, **poly14** was insoluble in organic media, and was therefore unable to be characterized. **Poly14**–**poly18** displayed *T*_d_ values ranging from 275–331 °C, which was considerably higher than the other analogues of the series. The polymers had *T*_g_ values ranging from −1 to 79 °C, while the absence of detectable *T*_m_ values was attributed to atacticity. Stereoregular samples of **poly15** were then produced from chiral starting materials. The stereoregular polymers exhibited *T*_m_ values of 154 °C on the first heating cycle, which was absent during the second cycle, consistent with previous reports for the series. An equimolar blend of the two isotactic polymers **poly**(***S***)-**15** and **poly**(***R***)-**15** gave a stereocomplexed material which had a *T*_m_ value of 175 °C during both heating scans. A distinct crystallization cooling peak was also observed which indicated that stereocomplexation could be used to accelerate the crystallization process.

Depolymerization studies were conducted in both solution and in bulk to evaluate chemical recyclability. When 2 mol % **cat8** was applied in dilute toluene solution (0.02 M) at 120–140 °C, **poly15** and **poly16** were readily depolymerized with monomer recovery yields of 78% and 94%, respectively. However, **poly17** was reported to only provide 42% monomer recovery, alongside significant quantities of side product. Interestingly, **poly18** gave quantitative depolymerization, which was speculated to be due to the geminal dimethyl group accelerating ring-closure. Bulk thermal depolymerization was then performed at 115–150 °C using Sn(AcO)_2_ as a catalyst. Under these conditions, monomers **15**, **16**, and **18** were recovered in yields of ca. 97%, and even **poly17** was reported to readily depolymerize to give a monomer yield of 93%, without side reaction.

**Figure 14:**
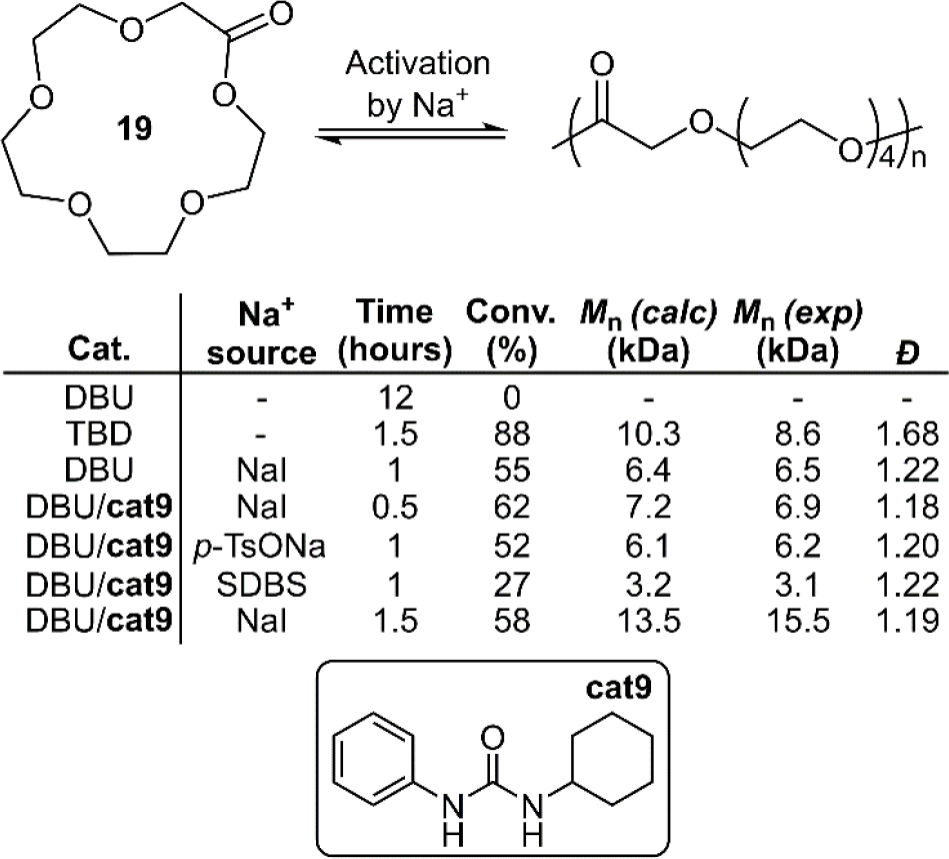
Polymerization conditions: [M]/[Na^+^]/[Cat]/[BnOH] ratio of 50/1.25/1.25/1, respectively, in DCM at 25 °C.

The polymerization of macrocyclic ester **19** was recently reported by Chen et al. for the preparation of PEG-like polyesters (Figure 14).^45^ Typically, lactones with over 12 atoms have low ring-strain and are therefore less prone to polymerization. While DBU failed to catalyze the ROP of **19**, TBD was reported to promote uncontrolled ROP. The coordination between cations and crown ethers has been widely reported in supramolecular chemistry and is perhaps able to enhance the electrophilicity of the carbonyl group of a lactone monomer, and thus an approach involving the activation of **19** by Na^+^ cations was developed. Although the application of DBU alone failed to produce polymer, the addition of NaI was reported to provide controlled polymerization to give **poly19**. Sodium *p*-toluenesulfonate (*p*-TsONa) and sodium dodecylbenzenesulfonate (SDBS) were also evaluated for their activation ability, but were reported to be inferior. The addition of urea cocatalyst **cat9** to the DBU/NaI system was reported to improve both the molar mass and dispersity. Unfortunately, higher monomer conversions (≥75%) were reported to generate higher dispersities. This effect was explained by the inherent binding selectivity of the Na^+^ cations to **19** in comparison to **poly19** being compromised by higher quantities of ring-opened products, i.e., increased cation binding to **poly19** promoting trans-esterification. This effect was later exploited for the depolymerization of **poly19** by the application of DBU and a higher quantity of NaI. ^1^H NMR analysis indicated that the major depolymerization product was **19** (>90%), with the remainder being the cyclic dimer of **19** which was reported recyclable by employing vacuum distillation.

**Figure 15:**
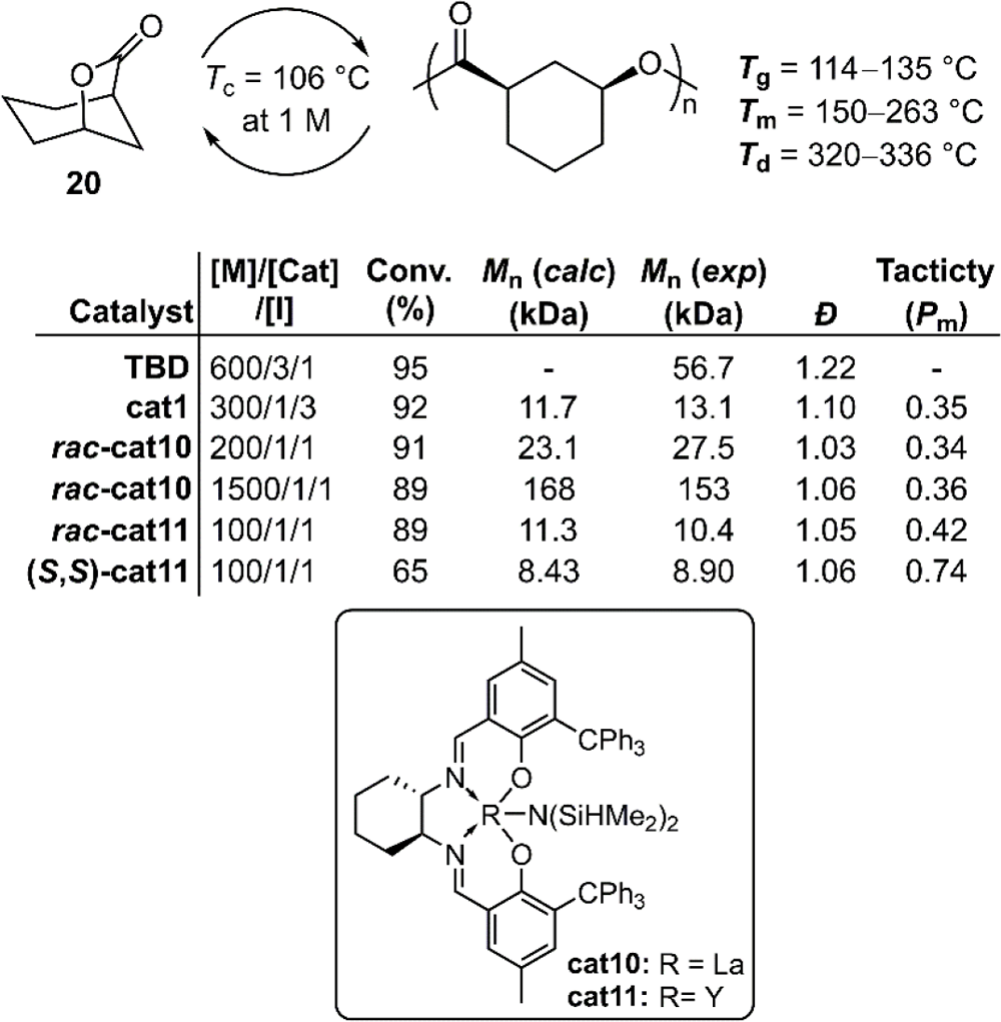
Polymerization conditions: 6 M in toluene at 25 °C; initiator (I) = BnOH.

The fusion of a high *T*_c_ substructure for polymerizability with a low *T*_c_ substructure for depolymerizability into a single monomer was realized by the fusion of ε-caprolactone and γ-butyrolactone, respectively, to give monomer **20**.^46^ Remarkably, **poly20** was reported to have a *T*_g_ value of ≤135 °C and a *T*_m_ value of ≤263 °C, which are approximately 200 °C higher than those of the parent polymers. **Poly20** exhibits two stereogenic centers per repeating unit which provided the researchers with an opportunity to target specific tacticities to thereby tune material properties. To this end, multiple catalysts and conditions were examined. TBD was first applied as a catalyst and benzyl alcohol as an initiator at 25 °C with a [M]/[TBD]/[BnOH] ratio of 600/3/1, respectively, providing 95% conversion and a *M*_n_ = 56.7 kg mol^–1^ (*Đ* = 1.22) (Figure 15). Analysis by NMR revealed that epimerization occurred during the ROP, giving both *cis* (82%) and *trans* (18%) stereoconfigurations. As a result, the resulting polymer was an amorphous material which displayed a *T*_g_ of 119 °C.

To suppress epimerization, a variety of coordination–insertion polymerizations were examined using lanthanum and yttrium complexes (**cat10**/**cat11**, respectively) (Figure 15). These catalysts were reported to provide polymer with entirely *cis*- configuration. Chiral forms of these catalysts (i.e., (***S***,***S***)-**cat11**) provided semicrystalline polymers which were evidenced by *T*_m_ values (>150 °C). Furthermore, isotactic **poly20** with a *M*_n_ = 19.3 kg mol^–1^ (*Đ* = 1.08) was prepared from **(1*R*,5*S*)-20** using **cat1**, which exhibited a *T*_m_ of 263 °C but no observable *T*_g_. Samples of **poly20** were then examined for their depolymerizability. The application of TBD as a depolymerization catalyst was reported to generate further epimerization, followed by the full depolymerization of both *cis* and *trans* chains within 12 h. Depolymerization using **cat1** was reported to achieve near completion (>95%) after heating at 120 °C for 36 h.^46^

### 2.3 Cyclic Thioesters

**Figure 16:**
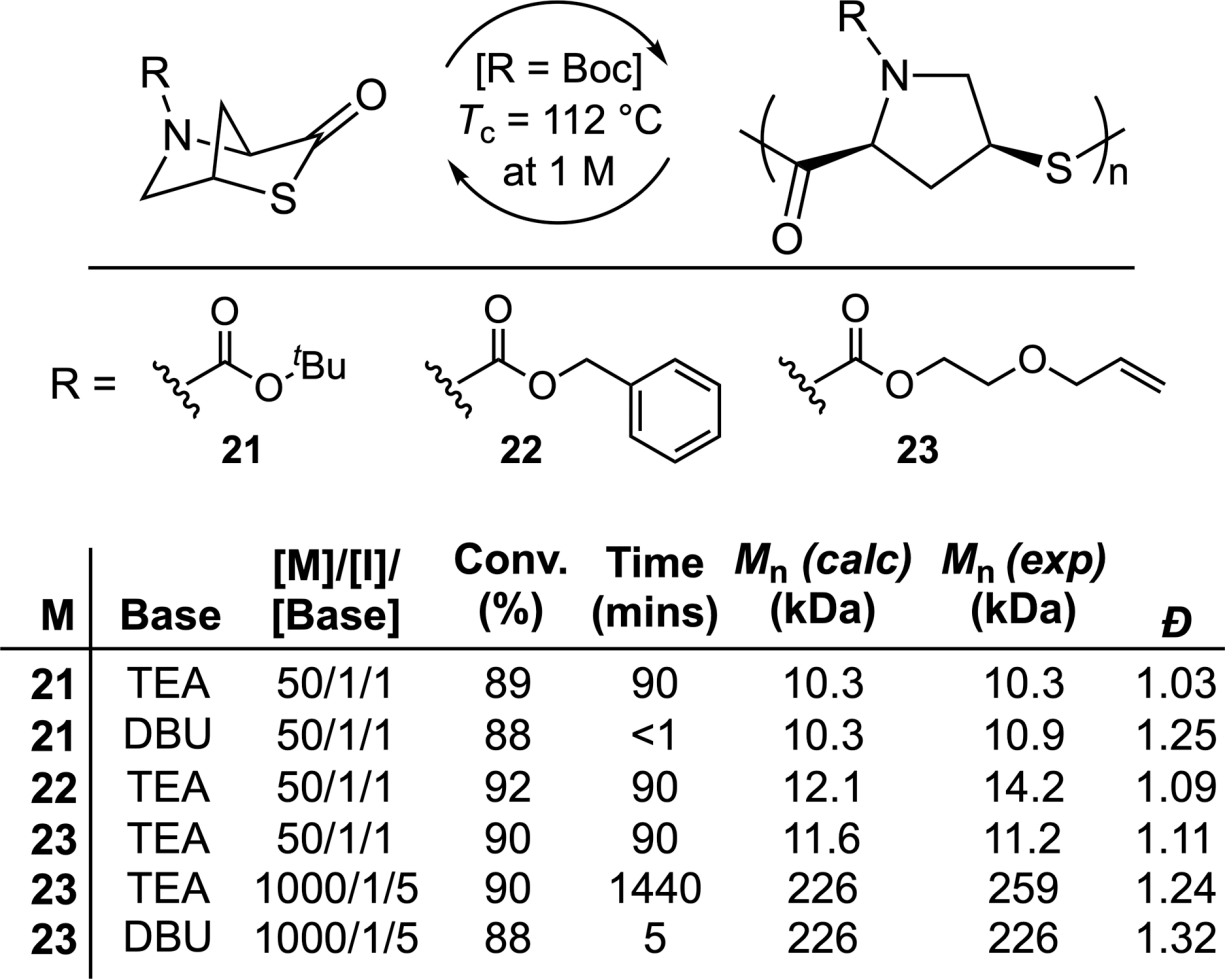
Polymerization conditions: 2 M in CDCl_3_ at 25 °C using benzyl mercaptan.

Three monomers (**21**–**23**) derived from *trans*-4-hydroxy-l-proline, a biorenewable feedstock, were examined by Lu et al.^47^ (Figure 16). Boc-substituted analogue **21** was reported to be readily polymerizable using benzyl mercaptan as a catalyst and TEA as a base. A change of catalyst to DBU was reported to provide significantly faster polymerizations at the expense of slightly worse dispersities. The copolymerization of **21** and **23** was also performed by sequential addition, successfully providing a block copolymer. **Poly23** (*M*_n_ = 11 kg mol^–1^) was reported to have a *T*_d_ of 198 °C and a *T*_g_ of 32–37 °C. **Poly22** had a *T*_g_ of 67 °C, whereas there was no measurable glass transition for **poly21**. None of the polymers displayed a *T*_m_. To examine their chemical recyclability, a sample of **poly21** was reacted with TEA (4.6 equiv. relative to repeating units) in dilute chloroform, providing a temperature dependent depolymerization. When a catalytic amount of DBU (0.046 equiv) was applied at 50 °C, quantitative depolymerization was realized within 2 min. Analysis by SEC suggested that depolymerization occurred in a domino-like fashion, without random chain-scission. Moreover, thermolysis of **poly21** under vacuum also provided 79% monomer recovery.

**Figure 17:**
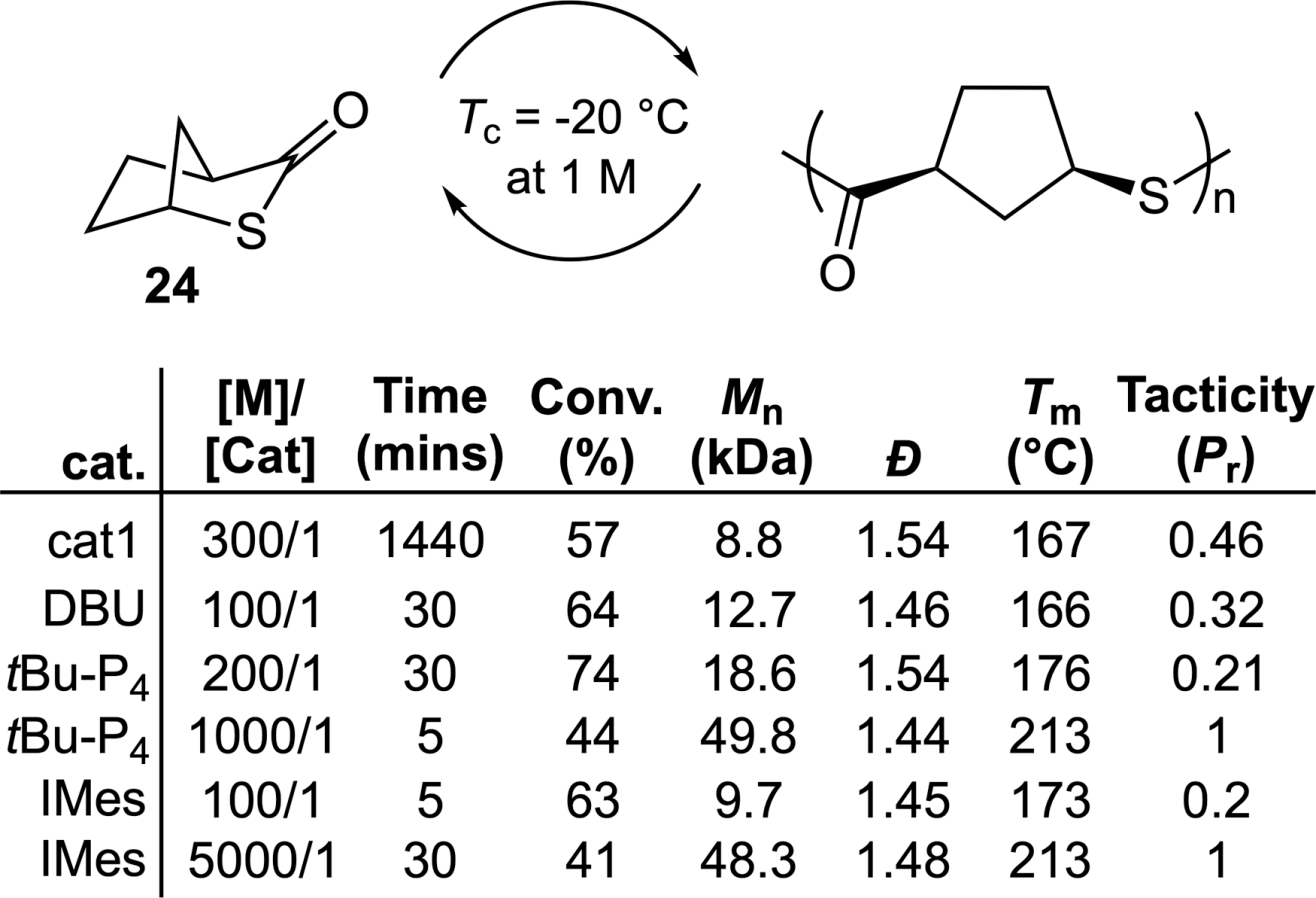
Polymerization conditions: 1 equiv of BnOH relative to catalyst, or 3 equiv relative to **cat1**, in toluene at 25 °C.

As monomers **21**–**23** had low *T*_d_ values (*ca*. 200 °C) and no measurable *T*_m_ which indicated a lack of crystallinity, Chen et al. therefore examined the ROP of bicyclic monomer **24** (Figure 17).^48^ The ROP of **24** using **cat1** at 25 °C at a [M]/[Cat]/[BnOH] ratio of 300/1/3, respectively, gave only 57% conversion after 24h, with the resulting polymer reported to be nonstereoregular. Similarly, DBU provided an atactic but semicrystalline material with a *T*_m_ value of 166 °C. The use of superbase phosphazene *t*Bu-P_4_ afforded a mostly atactic polymer with a comparatively higher *T*_m_ value of 176 °C. Interestingly, when monomer concentration was increased and catalyst loading decreased, a stereoregular polymer was obtained with a *T*_m_ of 213 °C and a *T*_g_ of 112 °C. Moreover, it was reported that when *N*-heterocyclic carbene (NHC) IMes was applied as a catalyst, crystalline samples of **poly24** could also be prepared. In this way, crystallinity could thereby be tuned, providing polymers with *T*_m_ values ranging from 166–213 °C, with this value being directly related to tacticity which modulates crystallinity. Bulk depolymerizations of **poly24** were reported to be facile at 100 °C in the presence of catalytic quantities of **cat1**, giving >90% isolated monomer yield after 24 h. Solution depolymerization in toluene (2 M) using IMes (2.3 wt %) at 25 °C were reported to give quantitative depolymerization after only 10 min. As a proof of concept, a sample of recycled **24** was also successfully repolymerized to **poly24**.

**Figure 18:**
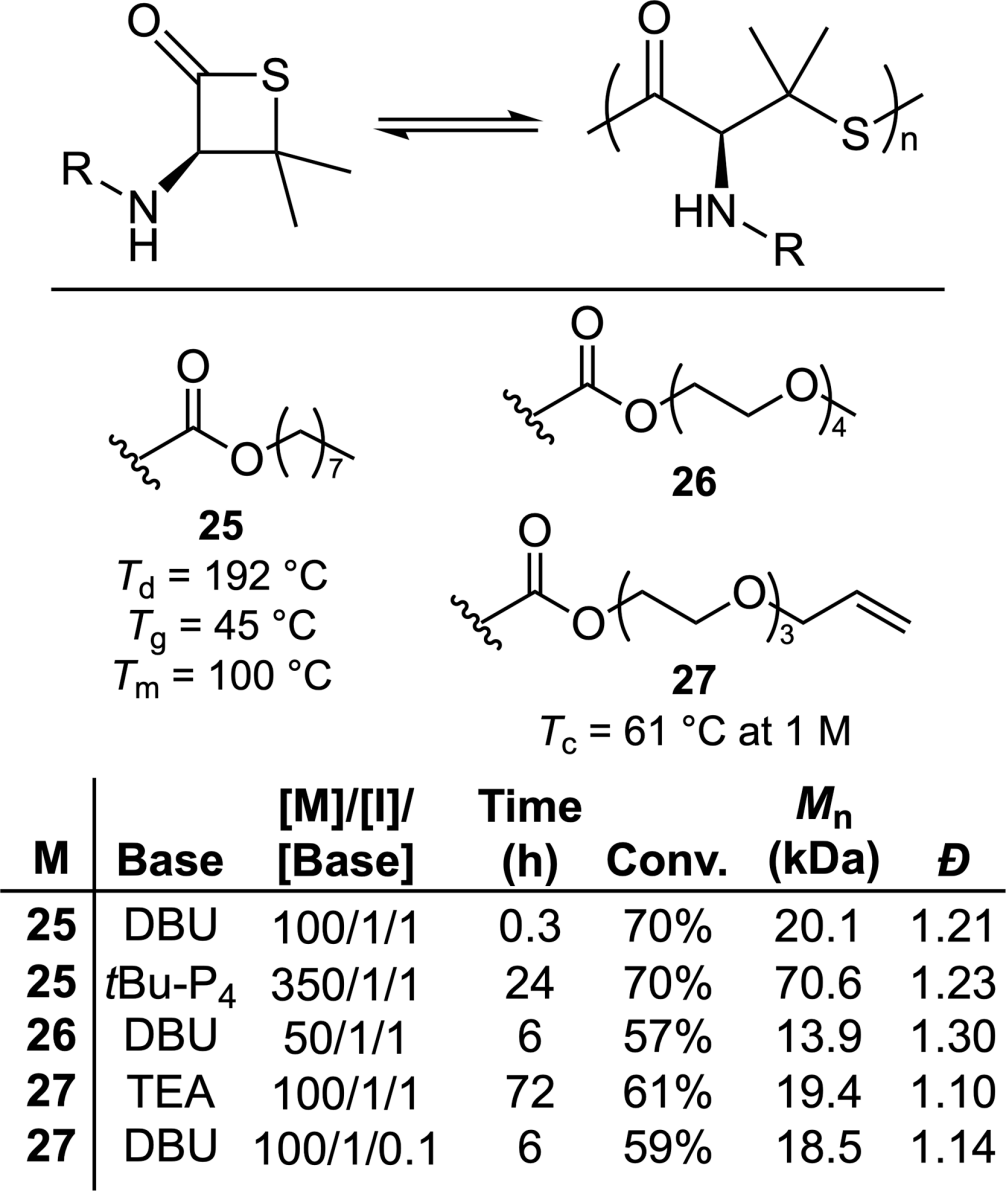
Polymerization conditions: bulk using benzyl mercaptan as an initiator at 25 °C.

The introduction of geminal dimethyl groups is a well-known approach for accelerating ring-closure by the so-called Thorpe–Ingold effect (e.g., **poly18**). Accordingly, a series of monomers (**25**–**27**) were synthesized from naturally occurring amino acid d-penicillamine (Figure 18).^49^ Their ROP was initiated by benzyl mercaptan and catalyzed by an organobase. When the ROP of **22** was catalyzed by TEA, **poly27** with a *M*_n_ value of 19.4 kg mol^–1^ (*Đ* = 1.10) was produced. Substituting TEA with the stronger base DBU (0.1 equiv) accelerated the ROP from 72 to 6 h while preserving controllability as evidenced by a low dispersity and linear relationship between monomer conversion and *M*_n_. **Poly25** produced using phosphazene superbase *t*Bu-P_4_ (*M*_n_ = 70.6 kg mol^–1^) was reported to have a *T*_g_ of 45 °C, and *T*_m_ of 100 °C. The depolymerizability of **poly25** was examined using 0.05 equiv of DBU (relative to number of polymer chains) in THF at 65 °C, with a gradual regeneration of monomer observed. Examination by SEC indicated that no oligomers were formed during this process, thereby indicating that depolymerization occurred in an “unzipping-type” fashion. Unusually, **poly25** was reported to depolymerize into a racemic mixture at high temperatures (65 °C), but gave enantiopure **25** at reduced temperatures (i.e., 25 °C).

**Figure 19:**
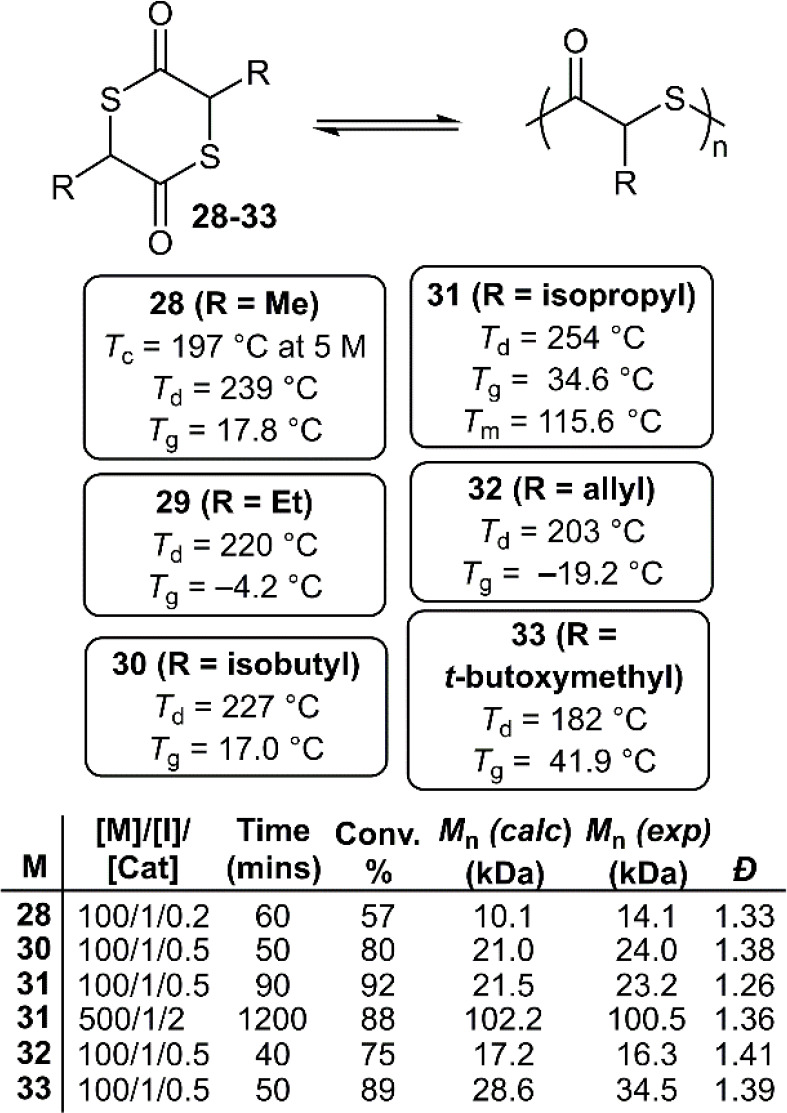
Polymerization conditions: performed using DMAP and benzyl mercaptan in DCM at 25 °C.

In 2021, a series of sulfonated analogues of lactide, the precursor to commercially available poly(lactic acid), were evaluated by Wang et al. (Figure 19).^50^ It was envisioned that an oxygen-to-sulfur substitution could accelerate ring-closure during depolymerization due to the decreased ring-strain, as well as provide polymers with enhanced material properties. It was reported that the ROP of *rac*-thiolactide (**28**) using DMAP at 25 °C led to a rapid and controlled polymerization. Moreover, it was reported that the *M*_n_ of the polymers increased linearly with the [M]/[I] ratio, giving unimodal distributions and low dispersities (*Đ* < 1.4). However, examination by MALDI-TOF revealed that *trans*-thioesterification was occurring during polymerization.

The scope was later expanded to include a variety of analogues (**29**–**33**). Their ROP provided a polymer series with *T*_g_ values ranging from −19.2 to 34.6 °C, demonstrating a significant influence of the side chains. Unusually, **poly31** (R = isopropyl) was determined to have a *T*_m_ of 115.6 °C which, alongside XRD measurements, demonstrated that it was an atactic yet semicrystalline polymer. **Poly31** with *M*_n_ values of ≤100 kg mol^–1^ (*Đ* = 1.36) could be synthesized using a [M]/[I]/[Cat] ratio of 500/1/2, respectively. To examine depolymerization, **poly28** was reacted with 1 mol % DBU (relative to repeating units) in dilute chloroform at 25 °C, giving quantitative monomer recovery within minutes. Depolymerization was reported to proceed in high selectively, giving a mixture of *rac*- and *meso*-thiolactide in a *ca*. 98:2 ratio which could be separated by recrystallization to provide ***rac***-**28**. Bulk depolymerization using vacuum distillation was also examined at 95 °C using catalytic amounts of DMAP, returning monomer in over 90% isolated yield.

### 2.4 Cyclic Amides

**Figure 20:**
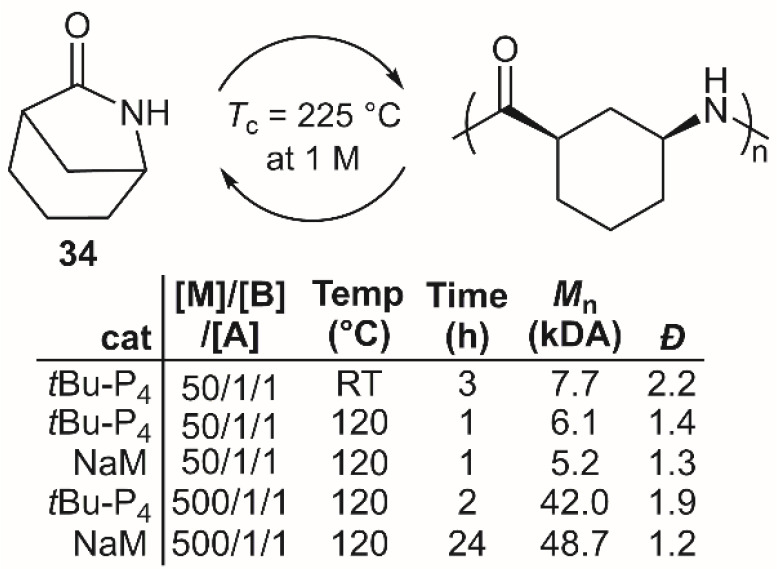
Polymerization conditions: performed in *N*-methylpyrrolidone (3 M) using *N*-benzoyl-34 as an activator [A]. NaM = Sodium adduct of the monomer.

Recently Chen et al. reported chemically recyclable amide **34**, which can be considered the fusion of the precursor compounds of nylon-6 (ε-caprolactam), a high *T*_c_ polyamide, and nylon 4 (pyrrolidone), a low *T*_c_ polyamide that is chemical recyclable but thermally unstable (Figure 20).^51^ It was reported that **34** could be polymerized by anionic ROP by employing a strong base catalyst and an *N*-acyl substituted activator derived from the monomer. It was described that the use of the sodium adduct of the monomer (NaM) offered the highest molecular weights and lowest dispersities, although it required comparatively more time to achieve high yields. Depolymerization of **poly34** by heating to 290 °C with the addition of ZnCl_2_ to activate the carbonyl groups for amine backbiting resulted in 93–98% recovery of **34** in reasonable purity.

**Figure 21:**
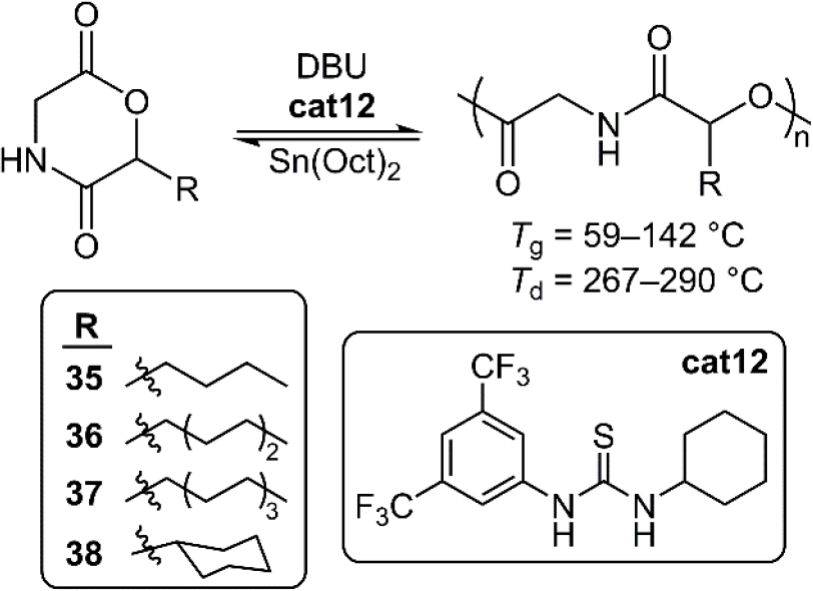
(De)polymerization of various morpholine-2,5-dione analogues.

In two publications, Li et al. demonstrated the (de)polymerization of morpholine-2,5-dione analogues (**35**–**38**, among other analogues) (Figure 21).^52,53^ It was reported that polymerizations applying DBU or TBD alone were not well-controlled, hypothesized to be due to deprotonation of the amide moiety. To prevent this, **cat12** was employed alongside DBU (5:1 ratio, respectively), affording significantly improved controllability. While *n*-alkyl substituted monomers **35**–**37** all had similar kinetic profiles, monomer **38** was reported to have a drastically slower polymerization rate which was ascribed to steric hindrance of the cyclohexyl group. Measurements by DSC revealed that all polymers in this series were amorphous owing to the irregular stereochemistry of the side-chains. The *T*_g_ values of **poly35**–**poly37** were in the range of 58–81 °C, influenced by both the side-chain length and *M*_n_ value. Meanwhile, **poly38** (*M*_n_ = 13.8 kg mol^–1^) possessed a high *T*_g_ of 142 °C which could be explained by the combined effects of a sterically hindered substituent coupled with H-bonding interactions. Bulk depolymerizations were examined using Sn(Oct)_2_ as a catalyst and PEO as a reaction medium. Recovery yields of >90% were obtained in high purity, with even copolymers of **35**–**38** being reported to depolymerize efficiently.

### 2.5 Cyclic Acetals

**Figure 22:**
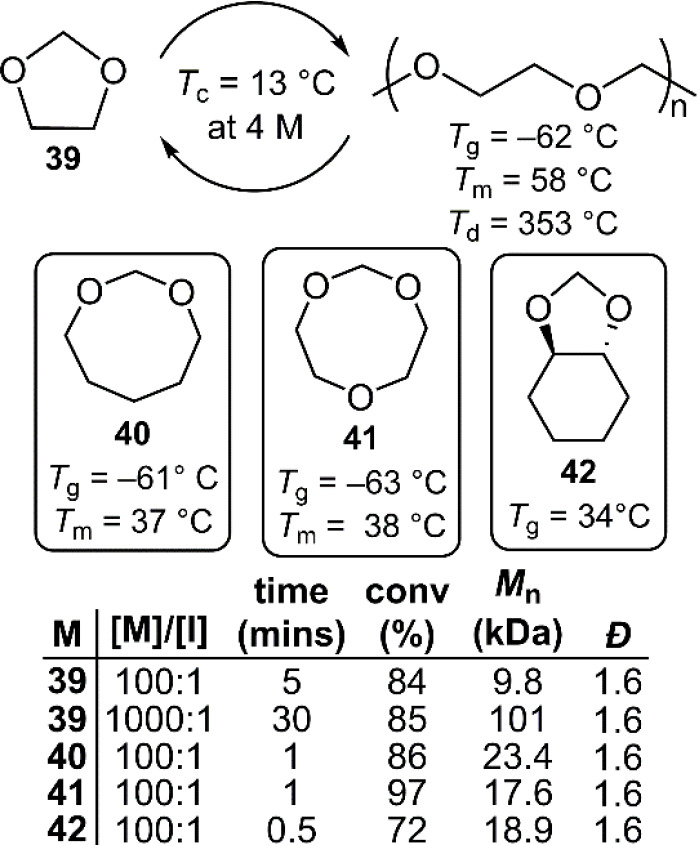
Polymerization were performed in CH_2_Cl_2_ at 25 °C; initiator (I) = MOMBr. [I]/[InBr_3_]/[DTBP]/[M] = X:0.5:5:300. DTBP = 2,6-di*tert*-butylpyridine, a proton trap.

In 2021, a seminal publication by Coates and co-workers reported the (de)polymerization of 1,3-dioxolane (**39**), which can be synthesized on large scale from formaldehyde and ethylene glycol, both of which are common feedstocks with bioderived routes (Figure 22).^17^ Controlled cationic ROP of **39** was achieved by the introduction of halide-terminated chain-ends by the addition of initiator bromomethyl methyl ether (MOMBr). This halide end-group can be reversibly deactivated in the presence of a Lewis acid catalyst, for which InCl_3_ and ZnCl_2_ were reported to be effective, with InCl_3_ offering better yield. Despite its lower activity, for commercialization purposes Zn-based catalysts may still be considered. The cationic ROP of analogues **40**–**42** using the InBr_3_/MOMBr system was also reported successful. Though excellent molecular weight control and livingness of the system were demonstrated, relatively high dispersity values of ca. 1.6 were obtained in all cases owing to transacetalization reactions, a known phenomenon during cationic ROP of cyclic acetals.

To examine the depolymerization of **poly39**, it was subjected to doping with 2 mol % camphorsulfonic acid and solvent-cast to ensure a homogeneous distribution. After heating at 140 °C at ambient pressure, pure **39** could be collected by distillation with ca. 98% monomer recovery. It was demonstrated that the recovered **39** could be dried over CaH_2_ and repolymerized to provide **poly39** with identical properties as the parent material. In addition, solid articles of **poly39** were mixed with articles of assorted postconsumer plastic waste and an acidic resin (Dowex-50, 5 wt %) and held at 150 °C to provide clean monomer by distillation, demonstrating that plastic separation would be not a requirement for the chemical recycling of **poly39**, a key advantage over other systems.

### 2.6 Cyclic Carbonates

**Figure 23:**
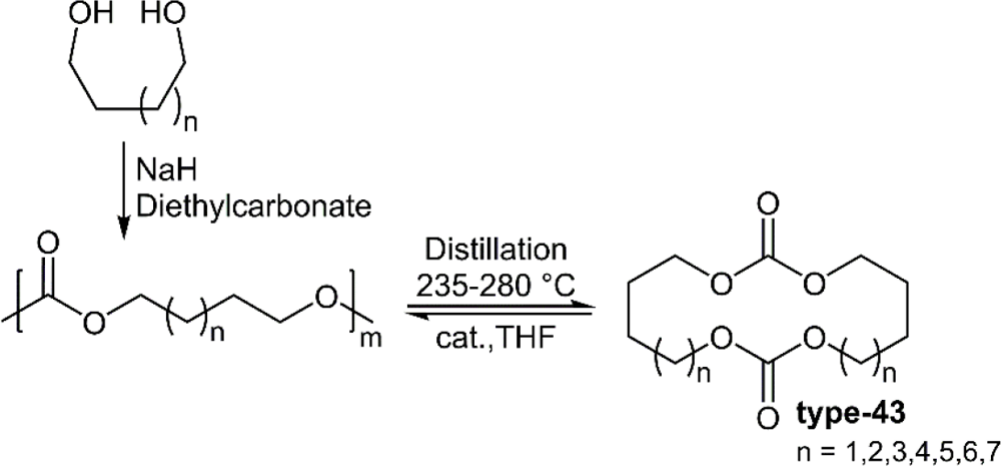
(De)polymerization of macrocyclic carbonates.

Recently, the Odelius group reported the synthesis and (de)polymerization of various macrocyclic carbonates (**type-43**, *n* = 1–7) (Figure 23).^54,55^ It was demonstrated that these macrocyclic monomers could be prepared by the ring-closing depolymerization of the corresponding polycarbonate, which could be readily produced with low *M*_n_ for this purpose by the polycondensation of diol compounds and diethylcarbonate. Once depolymerized to provide the corresponding cyclic carbonate monomer (**type-43**), these monomers can then be subjected to ROP by the application of BnOH and a catalyst to yield polycarbonate in high monomer conversion (>97%) and *M*_n_ (<53 kg mol^–1^) within seconds at 25 °C. Unusually, it was reported that the polymerization rate was dependent upon the odd or even number of methylene groups between the carbonate moieties, and not the overall ring size. The depolymerization of **polytype-43**_**ROP**_ were examined at 240–260 °C by vacuum distillation, with monomer recoveries (70–85%) reported to be higher than samples produced by polycondensation, theorized to be due to less side reactions such as alcoholysis and dehydration occurring during the ROP. A similar cyclic carbonate system involving a monomer to polymer ⇌ dimer system was reported by Zhu et al., albeit with a considerably less efficient (de)polymerization system.^56^

## 3 Ring-Opening Metathesis Polymerization (ROMP)

**Figure 24:**
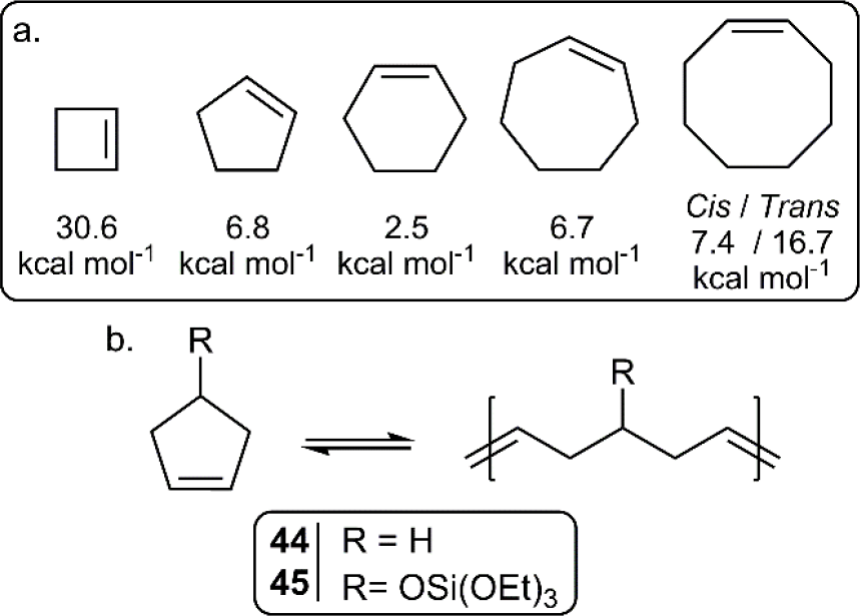
(a) Ring-strain energies (RSE) of cycloalkenes; (b) (de)polymerization of polypentenamers.

As olefin metathesis cannot occur without a catalyst, unintended depolymerization can be prevented by simply removing the catalyst from the system. Thereby, olefin metathesis can be considered an attractive approach for the preparation of chemically recyclable polymers. In comparison to ROP, ROMP provides relatively stable polymers that contain a hydrocarbon backbone without the addition of unnecessary heteroatoms, unless by design. Regarding the cycloalkene series, cyclohexene has the lowest ring-strain energy (RSE) at 2.5 kcal mol^–1^, which is too low for polymerization (Figure 24a).^57^ Owing to the high RSEs of cyclopropene and cyclobutene (54.5 and 30.6 kcal mol^–1^, respectively), their depolymerization is not realistic.

Cyclopentene (**44**), with an RSE of 6.8 kcal mol^–1^, was reported to be (de)polymerizable as early as 1972 (Figure 24b).^58^ Bazzi et al. later demonstrated that a silicon-functionalized analogue (**45**) could also be quantitatively depolymerized, proposing its use in recyclable tires.^59^ Moreover, various cyclopentane analogues with *T*_c_ values ranging from 50 to 100 °C were revealed as useful candidates for the preparation of networks that can be depolymerized, reshaped, and then reformed.^60^ In addition, analogues of **44** containing an ATRP initiator moiety have also been applied to create self-immolative bottlebrush polymers.^61^

**Figure 25:**
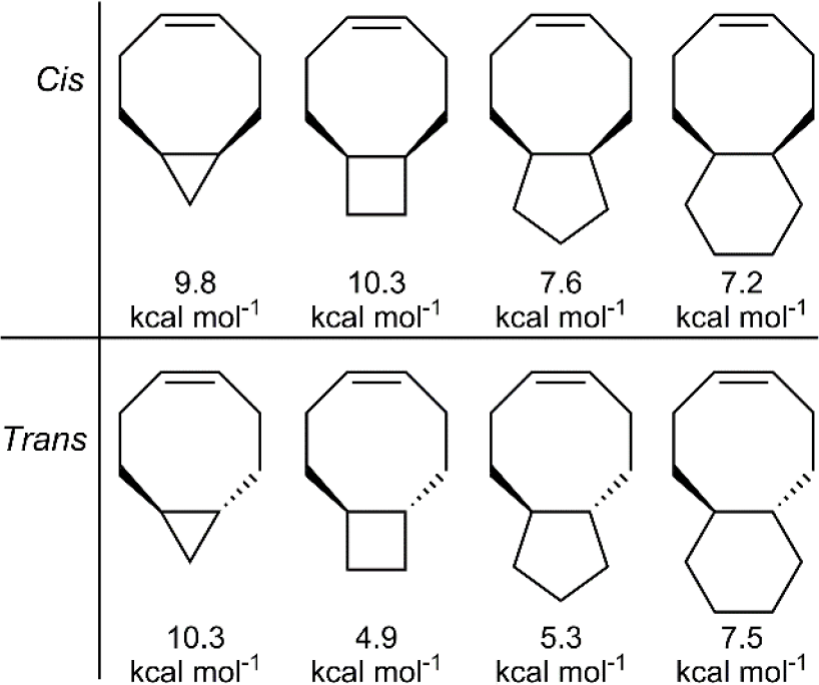
Ring-strain energies (RSE) of the cycloalkene and fused-cyclooctene series.

Cyclooctene can also be considered an attractive scaffold choice as there exists convenient synthetic access to many analogues, but its high RSE (7.4 kcal mol^–1^) would prevent its depolymerization. Computational calculations demonstrated that the fusion of a cyclobutane or cyclopentane ring at the 5,6-position of cyclooctene could lower the RSE to 4.9 or 5.3 kcal mol^–1^, respectively, thereby making these attractive monomer choices for ROMP (Figure 25).^16^ In addition, if necessary the *Z*-alkene of the cyclooctene could be isomerized to an *E*-alkene, significantly increasing the RSE.

**Figure 26:**
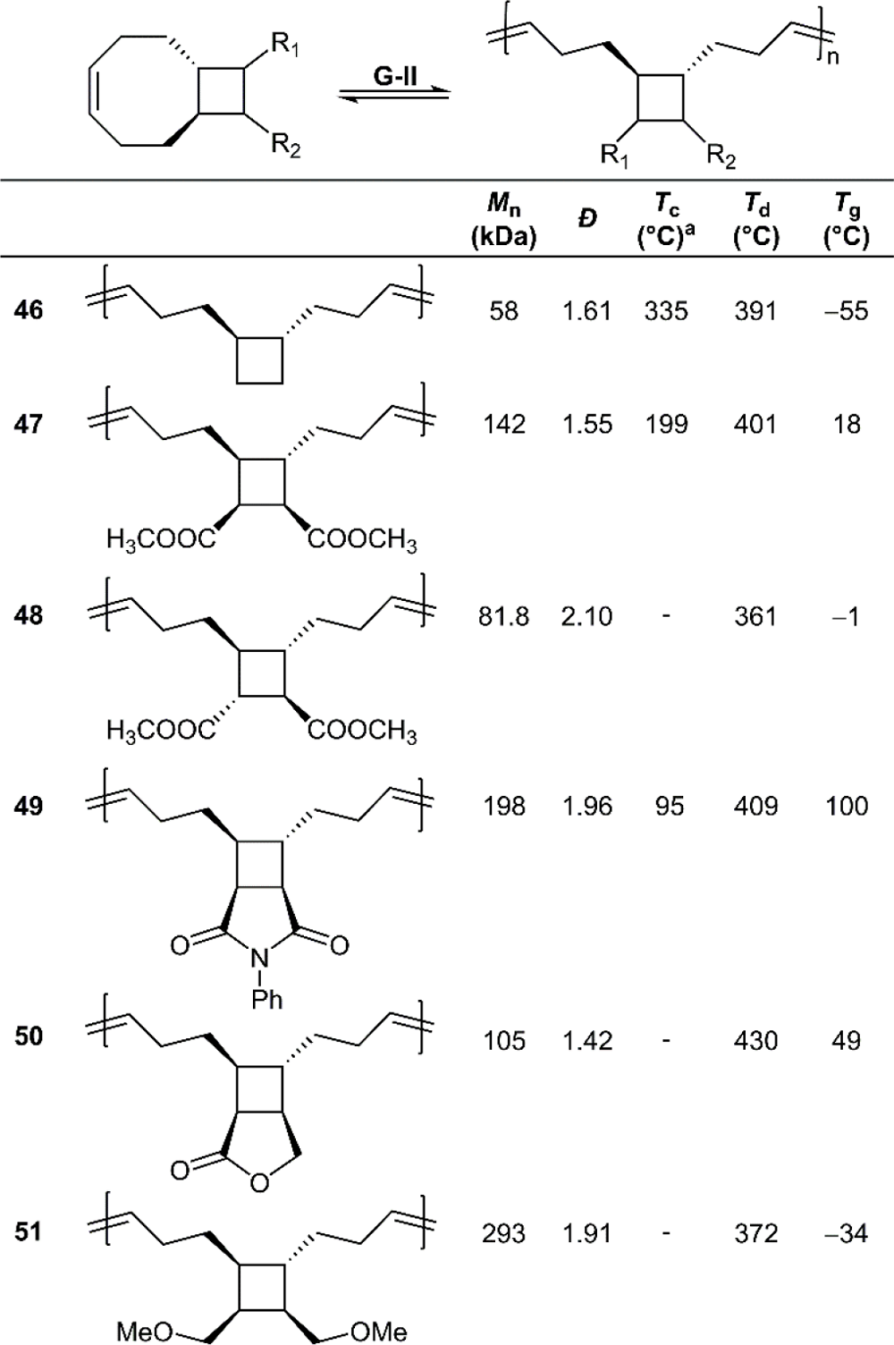
Polymerization conditions: ca. 2 M in DCM using G-II catalyst; ^a^ceiling temperature at 1 M monomer concentration.

A variety of cyclooctene monomers (**47**–**51**) were therefore prepared by the reaction of 1,5-cyclooctadiene and maleic anhydride, followed by further derivatization, while monomer **46** was synthesized in a more complex procedure (Figure 26).^16,18^ Fortunately, all syntheses provided the *trans*-fused analogue, which was the targeted molecular structures due to the comparatively lower RSE. Monomers **46**–**51** were readily polymerized at [M]_0_ = 2 M in DCM using second-generation Grubbs (G-II) catalyst to provide monomer conversions over 80% and *M*_n_ values of >100 kg mol^–1^. Substitution effects can be readily assessed by their effect on the *T*_g_ value, for example when the protons of the cyclobutane ring in **46** are substituted by methyl ethers (**51**), methyl esters (**47**), or a fused imide-containing ring (**49**), the *T*_g_ value changes from −55 °C for the parent material to −34, 18, and 100 °C, respectively. Moreover, when the *cis*-substituted analogue **47** is compared to the *trans*-substituted analogue **48** there exists a considerable difference in their *T*_g_ values (18 °C versus −1 °C, respectively).^62^ These phenomena therefore allow the tuning of material properties. To examine the ring-closing metathesis (RCM) depolymerization, the polymers were heated with 1 mol % G-II catalyst in chloroform at 50 °C. All polymers readily underwent depolymerization (>90%), with recovered **47** being repolymerized to give **poly47** with a *M*_n_ value = 71 kg mol^–1^ (*Đ* = 1.53). Upon careful SEC examination, it was concluded that the polymers were first depolymerized into oligomers by random chain cleavage.

**Figure 27:**
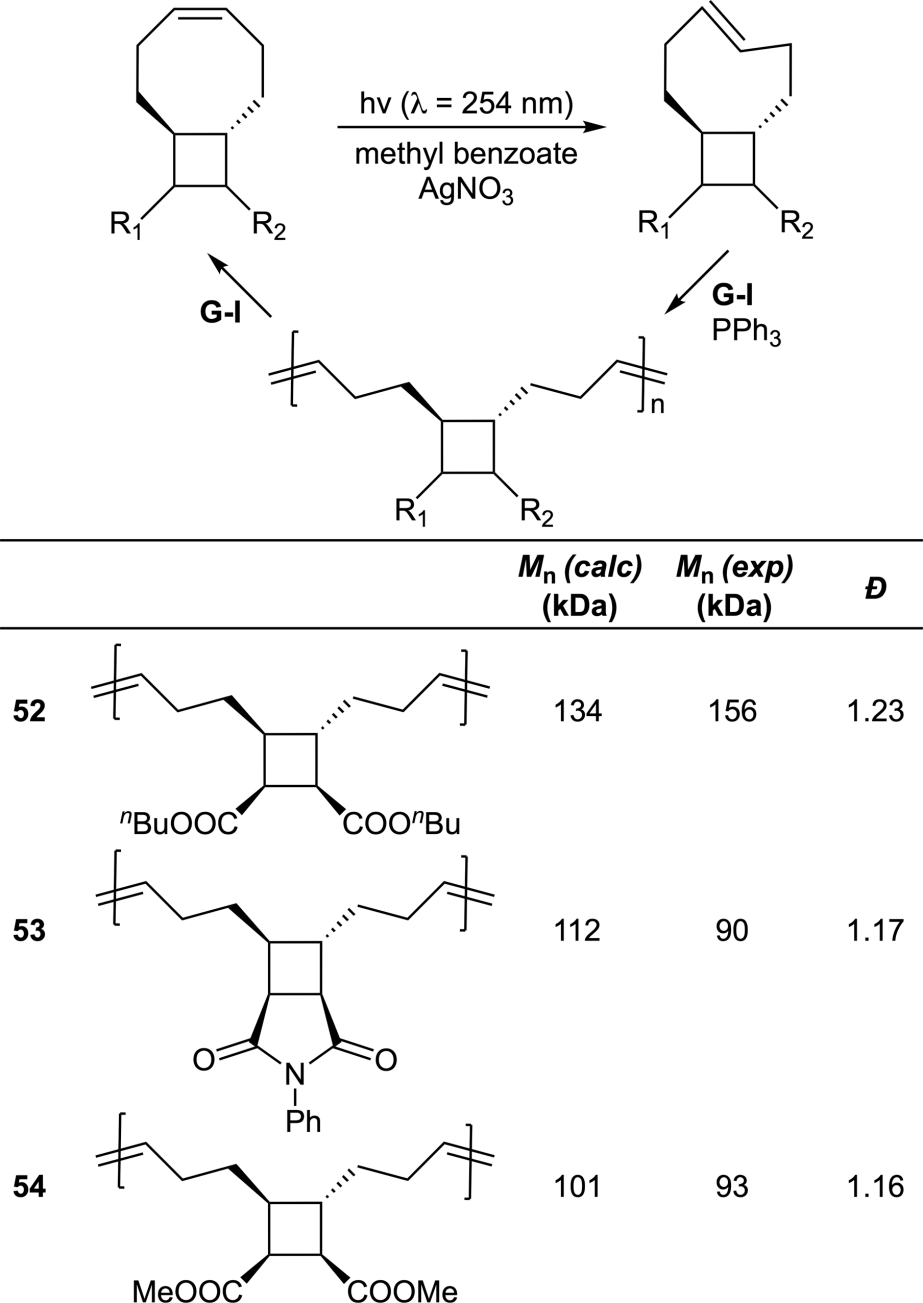
Polymerization conditions: ca. 0.25 M in THF using G-I catalyst with 60 equiv of PPh_3_.

As high monomer concentrations (>1.0 M) were reported essential for efficient polymerization, a new system was explored where monomers were first photoisomerized to the *E*-alkene and then applied to ROMP, giving excellent monomer conversions (>95%) at concentrations as low as ≥0.025 M(Figure 27).^63^ The isomerization from a low-energy (*Z*-alkene) to high-energy (*E*-alkene) state allows polymerization at lower monomer concentrations due to the higher RSE which provides a polymerization driving force.^16^ Polymer can be depolymerized to provide the low-energy state monomer, and the cycle repeated. The photoisomerization process involves irradiation in the presence of silver nitrate to selectively bind the *trans*-cycloalkene to give a water-soluble complex which can be isolated and demetalated in yields ranging from 30% (**53**) to 81% (**54**). Optimization concluded that the addition of excess triphenylphosphine was able to suppress ring-closing depolymerization, this phenomenon being readily identified by *Z*-alkene monomer in the NMR spectra. Moreover, a block copolymer of **poly52**-***co***-**53** was synthesized by consecutive monomer addition. The prepared polymers were reported to depolymerize efficiently to the low-energy monomer in the presence of first-generation Grubbs (G-I) catalyst, with kinetic studies revealing that substitution does not significantly affect depolymerization.

**Figure 28:**
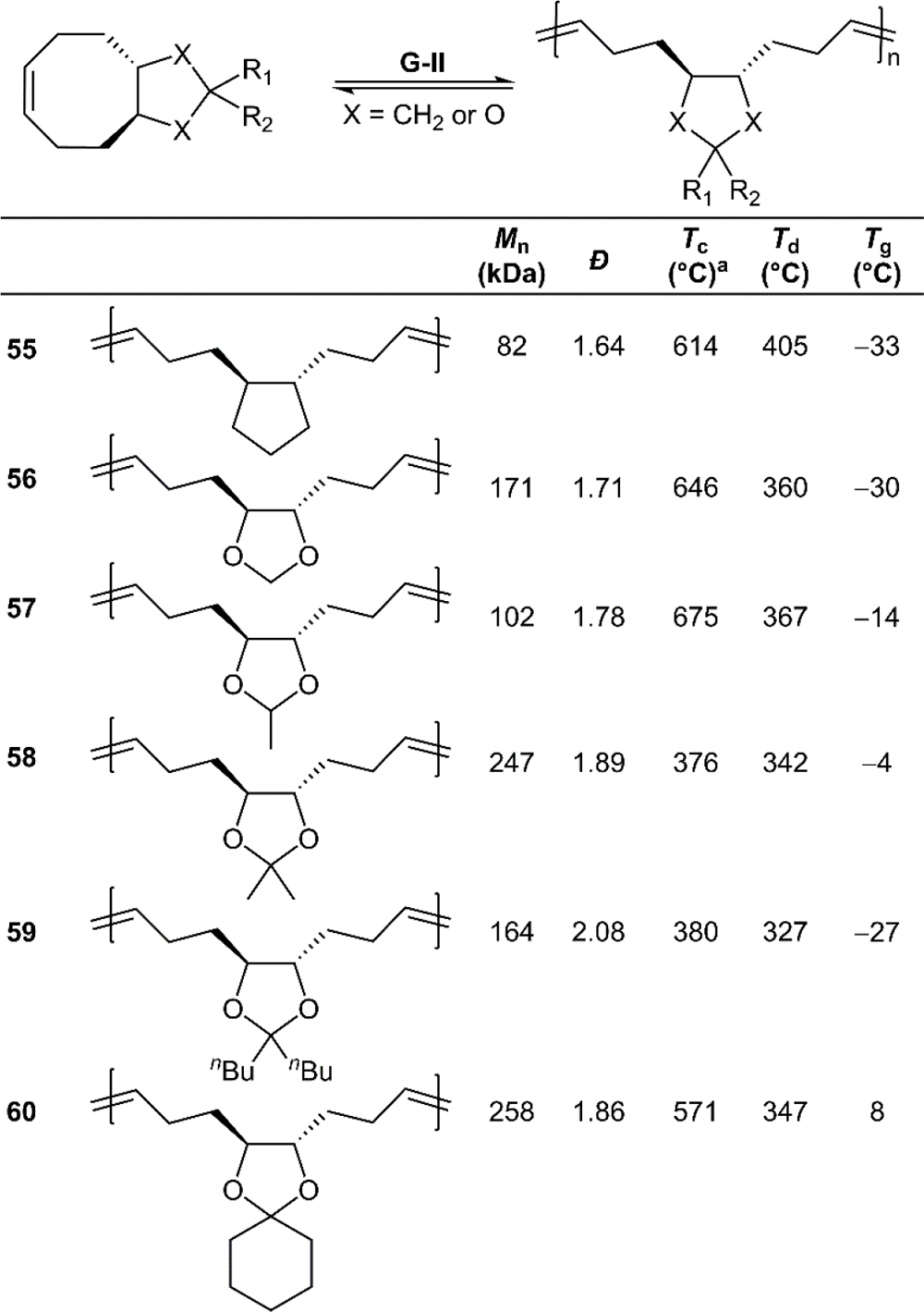
Polymerization conditions: ca. 2 M in DCM using G-II catalyst; ^a^ceiling temperature at 1 M monomer concentration.

Additional analogues containing a cyclic acetal moiety were later evaluated (**56**–**60**) (Figure 28). Curiously, there was no significant change in the ceiling temperatures between the cyclopentene fused analogue **poly55** (*T*_c_ = 614 °C) and its oxygenated counterpart **poly56** (*T*_c_ = 646 °C). The higher *T*_c_ value of the cyclopentene analogue **poly55** (*T*_c_ = 614 °C) in comparison to the cyclobutene analogue **poly46** (*T*_c_ = 335 °C) was reflected in its depolymerization behavior, leaving residual oligomer under conditions which quantitively depolymerized **poly46**. Compared to **poly56**, the introduction of a single methyl substituent in **poly57** (*T*_c_ = 675 °C) did not significantly alter the *T*_c_ value, although the introduction of an additional geminal methyl group in **poly58** significantly reduced the *T*_c_ to 376 °C. A change of the bis-methyl substitution to bis-*n*-butyl in **poly59** (*T*_c_ = 380 °C) did not significantly alter the *T*_c_. The addition of a fused cyclohexane made the *T*_c_ rise to 571 °C (**poly60**). These various phenomena thereby again offer evidence that geminal-disubstituion assists facile depolymerization.

**Figure 29:**
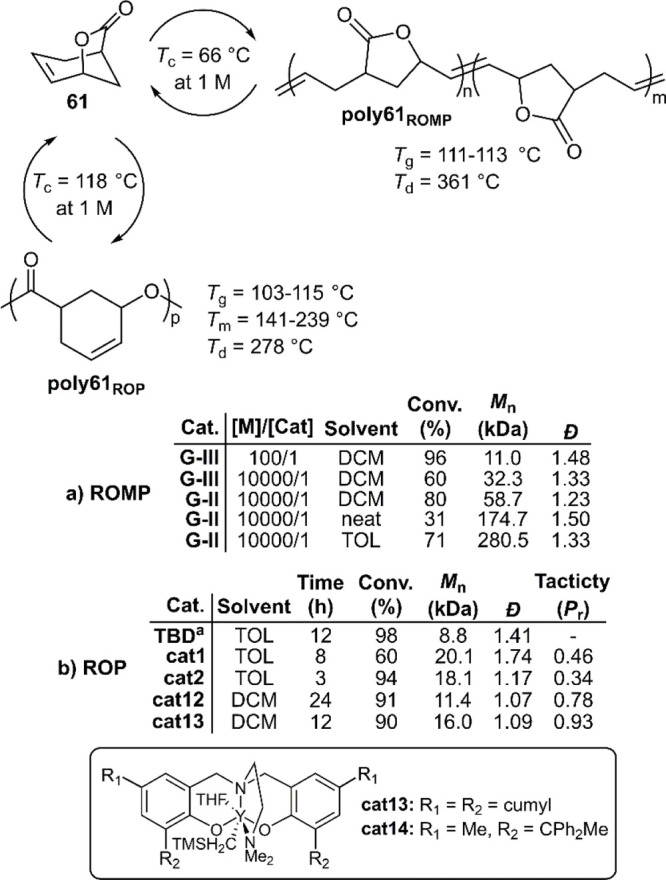
Polymerization conditions: (a) ROMP performed at 25 °C at a concentration of 5 M (or neat); (b) ROP performed at 25 °C at a concentration of 5–6 M and a [M]/[Cat] ratio of 100/1, respectively. ^a^Using 1 equiv of BnOH as an initiator, relative to the catalyst.

In an extraordinary report, Chen and co-workers described the synthesis of a monomer (**61**) which could be polymerized by either ROP or ROMP, depending on the catalyst, to give distinct polymers (Figure 29).^64^ It was reported that when the ROMP of **61** was performed using third-generation Grubbs (G-III) catalyst the polymerization proceeded rapidly at 25 °C, but that the measured *M*_n_ values deviated considerably from the calculated values. Next, G-II catalyst was examined and found to provide better control with catalyst loadings as low as 25 ppm, providing **poly61**_**ROMP**_ with *M*_n_ values as high as 340 kg mol^–1^ (*Đ* = 1.30). Unfortunately, the ROMP of an enantiopure sample of (***S***,***S***)-**61** revealed that the polymerization was not regioselective, and that *cis/trans* alkene configurations were both formed, thereby providing the same amorphous polymer as ***rac***-**61** with a *T*_g_ value of 111–113 °C.

For the ROP of **61**, TBD and **cat1** and were first screened for their catalytic ability (Figure 29). **Poly61**_**ROP**_ produced by TBD was reported to undergo epimerization at the stereogenic carbon to provide both *cis* (64%) and t*rans* (36%) stereoconfigurations, while **cat1** was reported to provide completely *cis*-configured polymer. In this way, a perfectly isotactic sample of **poly61**_**ROP**_ was produced by the polymerization of (***S***,***S***)-**61** using **cat1**, which had a high *T*_m_ value of 239 °C. To improve the stereoselectivity of the ROP of ***rac***-**61**, a variety of yttrium-containing catalysts were screened. **Cat2** was initially screened but was reported to provide an atactic polymer. A change of the catalyst ligand side arm from an β-OMe to β-NMe_2_ group was reported to significantly improve the selectivity. Thus, the application of **cat13** finally provided a semicrystalline sample of **poly61**_**ROP**_ as evidenced by a *T*_m_ value of 145 °C. Moreover, a switch to bulkier substituents to give **cat14** afforded **poly61**_**ROP**_ containing two *T*_m_ values of 141 and 195 °C.

The depolymerization of **poly61**_**ROMP**_ was reported to be facile using G-II catalyst, with a depolymerization demonstrated on a 28 g scale reported to give 93% monomer recovery. Conversely, the depolymerization of **poly61**_**ROP**_ was reported problematic. It was described that the depolymerization of **poly61**_**ROP**_ at high temperatures suffered from severe side-reactions, ascribed to the reactive alkene bond. Fortunately, the application of ZnCl_2_ in DCM at 40 °C was reported to give facile depolymerization. The authors examined a variety of conditions and concluded that the ZnCl_2_ catalyst was operating by an interfacial catalytic mechanism; i.e., depolymerization involved the particle surface. Monitoring the depolymerization reactions by GPC revealed that **poly61**_**ROMP**_ depolymerized by random chain scission, while **poly61**_**ROP**_ depolymerized by an “unzipping” mechanism. Moreover, a 1:1 physical blend of the two polymers was reported to depolymerize efficiently to monomer **61** when the catalysts were added in succession (G-II followed by ZnCl_2_).

**Figure 30:**
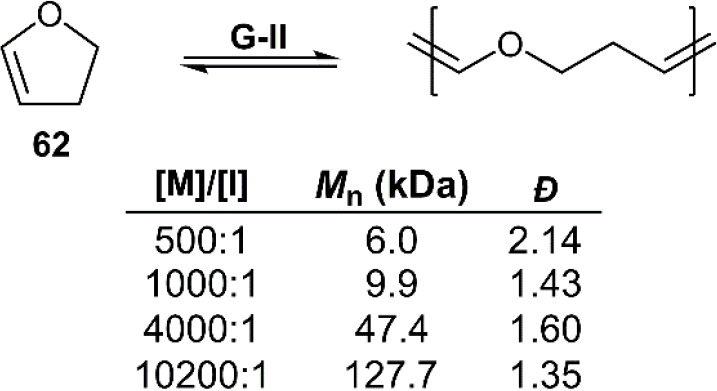
Polymerization conditions: neat using G-II catalyst at 25 °C for 72 h.

An additional ROMP monomer which displays promising chemical depolymerization behavior is 2,3-dihydrofuran (**62**) (Figure 30).^65^ Interestingly, such enol ethers are usually considered to be “quenching” reagents for olefin metathesis catalysts. Nevertheless, it was reported that **62** could be readily polymerized without solvent using G-I/G-II catalysts to produce a rubbery polymer with a *T*_g_ of −50.5 °C and *T*_d_ of 320 °C. The molecular weight of **poly62** was reported to vary linearly with catalyst loading, ranging from 6 to 127 kg mol^–1^ for a [M]/[I] value of 500/1 or 10200/1, respectively. To examine depolymerization behavior, a sample of **poly62** containing residual catalyst was heated for 2.5 h at 60 °C, giving *ca*. 90% monomer recovery by distillation, without side-reaction.

## 4 Conclusion and Outlook

Increasing quantities of postconsumer plastic waste are entering the environment, damaging fragile ecosystems and generating microplastic particles, highlighting the importance of developing next-generation polymers. The first step for confronting this issue is to address the fate of plastic waste. At present, most plastic waste is eventually incinerated, landfilled, or discarded directly into the environment. Furthermore, this is problematic from a sustainability perspective as only minimal value and energy is recovered. In recent years, chemically recyclable polymers have received increasing attention as a promising new strategy for addressing the plastic pollution problem. This approach can be considered especially attractive as the recovered monomer feedstock can be repolymerized to provide polymer with material qualities equal to that of virgin polymer, thus presenting a genuine closed-loop economy for plastic production. Moreover, chemical recycling eliminates the demand for new petrochemical feedstocks and also negates the energy required for monomer feedstock synthesis, offsetting other negative environmental effects.

To our best knowledge, at present, all reported chemically recyclable polymer systems have only been demonstrated on a bench scale, and thereby remain at a proof-of-concept stage. For a new polymeric system to be considered for commercial application it must demonstrate several factors including cost-effectiveness, material performance, and scalability. These requirements present a formidable challenge for polymer scientists. In addition, to develop a successful monomer/polymer system, a thorough understanding regarding thermodynamic polymerization equilibriums in regards to monomer structure and reaction conditions is required, for which the groundwork is presently being laid,^66−69^ including by computational approaches.^70^

A summary of the reported monomers and their attributes can be found in Table 1 (ROP monomers) and Table 2 (ROMP monomers). It can be readily observed that the cyclooctene-derived ROMP monomers (**46**–**60**) demonstrate an enormous variance in their attributes which can be tuned by relatively minor changes in the basic scaffold. Likewise, several ROP scaffolds have also exhibited significant property changes by minor molecular alterations (e.g., monomers **28**–**33**). One particular approach that could be leveraged for the modification of the *T*_c_ of reported systems is (geminal di)methyl substitution to accelerate ring-closure by the Thorpe–Ingold effect, with this effect already being noted for ROP monomers **18** and **25**–**27**, as well as ROMP monomer **58**. In addition, the substitution of heteroatoms into a cyclic scaffold can alter ring-strain due to their differing bond lengths and thereby influence the thermodynamics of (de)polymerization.^69^ A similar effect can be achieved by ring-fusion to a scaffold, as demonstrated for cyclooctenes (**46**–**60**) and γ-butyrolactone analogues (**2**–**4**). Moreover, the addition of bulky substituents at critical positions can be used to increase the *T*_g_ value by inhibiting the rotation of the polymer backbone.

**Table 1:**
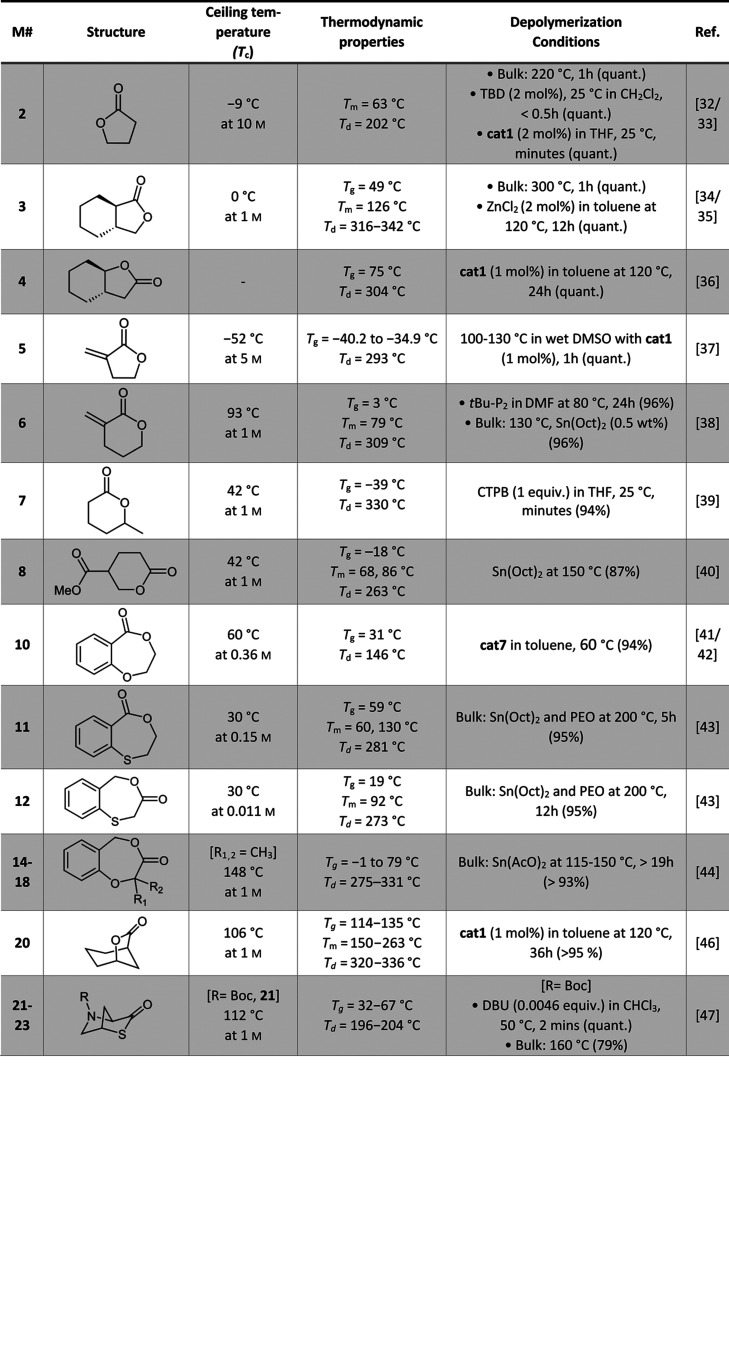

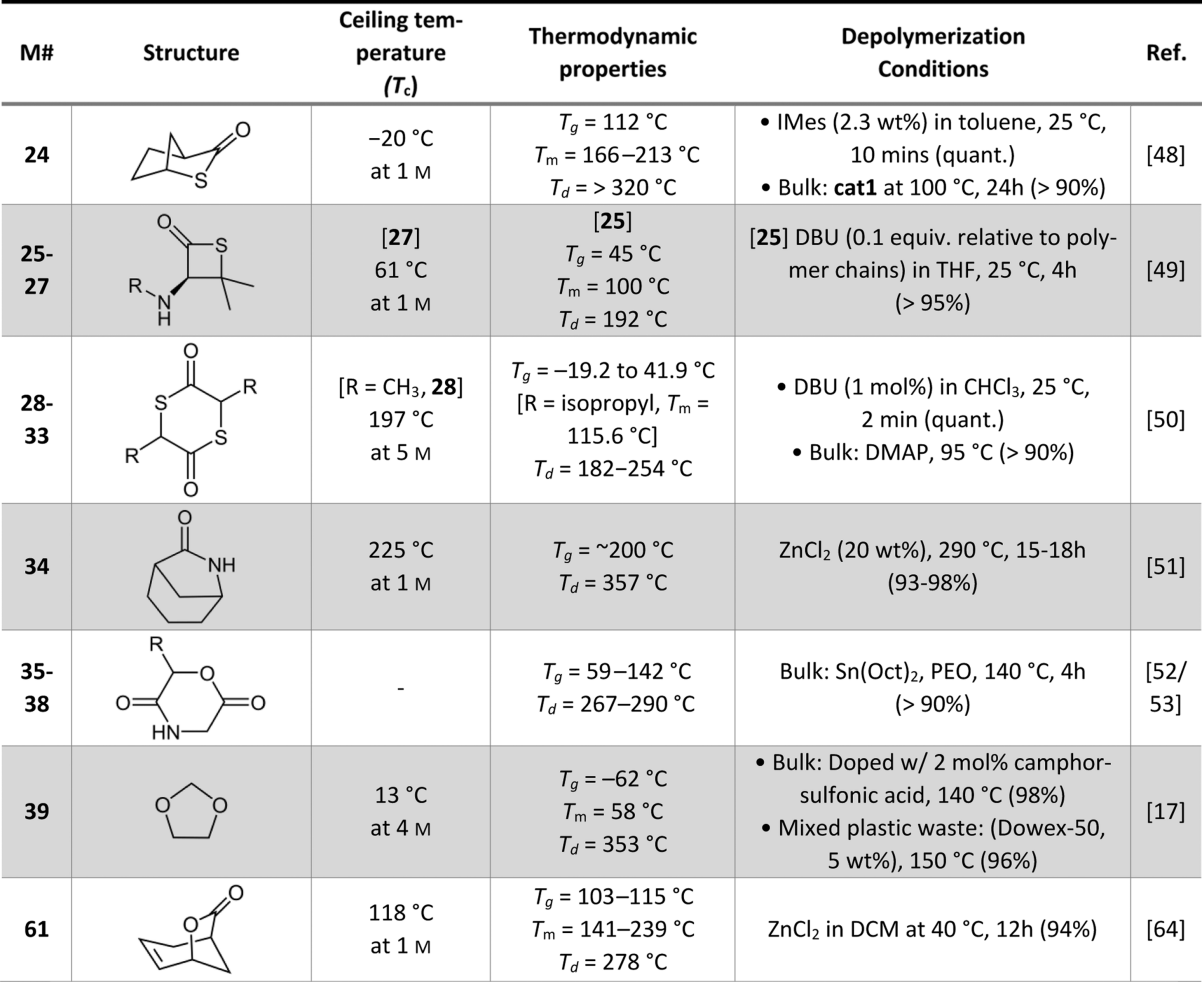
Summary of the Ceiling Temperature (*T*_c_), Material Properties, and Depolymerization Protocol and Yield for the Ring-Opening Polymerization (ROP) of Selected Monomers

**Table 2:**
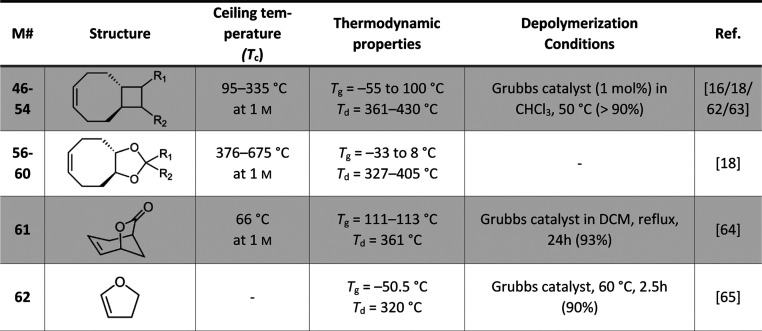
Summary of the Ceiling Temperature (*T*_c_), Material Properties, and Depolymerization Protocol and Yield for the Ring-Opening Metathesis Polymerization (ROMP) of Selected Monomers

It can be envisioned that future research will thereby aim to address the following four major challenges:

(1) Presently, the range of monomers that have been reported for chemically recyclable systems remains modest. More intensive research is required to identify new chemically recyclable systems that negotiate a balance between (de)polymerizability and material performance. Factors that need to be considered include selective depolymerization, reducing decomposition pathways, and that monomers have straightforward synthetic access. To streamline synthetic efforts, potential monomers could have their thermodynamic parameters computationally modeled before synthesis. For many of the presently reported approaches, there also exists prohibitive energy and material requirements which need to be addressed, for example, inert atmosphere and transition metal catalysts.

(2) For the commercialization of a new polymer system, another aspect that needs to be addressed is that the material properties need to compete with presently available commodity polymers. Several factors that need to fall within a desirable window include elongation at break, storage and loss moduli, tensile strength, gas permeability, melt flow, among many others. At present, difficulties related to thermal stability and poor material performance limit the widespread adoption of chemically recyclable polymers. It can even be envisioned that lower yields for the (de)polymerization processes could be offset by an exceptional polymeric system.

(3) The exploration of polymeric systems that maximize energy and thereby cost efficiency is also essential. Desirable systems are anticipated to have a finely tuned *T*_c_ value so that only minimal energy is required to perform depolymerization and, conversely, that the energy required for polymerization is also minimal. Due to the inclusion of additives such as catalysts and solvents, the design of an efficient purification strategy is also required. The exploration of purpose-driven catalysts for (de)polymerization can be considered a useful approach as minor variations in catalyst structure have been demonstrated to have dramatic effects on factors such as rate, topology, tacticity (therefore crystallinity), as well as *M*_n_ and dispersity. Moreover, the optimal catalyst for depolymerization can be expected to be a different catalyst than the optimal catalyst for polymerization, as these two processes are likely to be performed under vastly different conditions. For example, at the higher temperatures required for the depolymerization process, an effective polymerization catalyst may promote a competing reaction pathway, or even decompose.

(4) While a shift to a sustainable circular plastic economy is being undertaken, it would also be beneficial to consider any opportunities to incorporate “green” chemistry choices such as sourcing monomer feedstock from biobased and renewable resources. Other similar concepts involve the use of green catalysts, for example, reusable catalysts, organocatalysts, or catalysts incorporating nontoxic metals. The choice of solvent should also be given consideration, as multiple renewable and nontoxic options are presently available.

In conclusion, at present, the majority of chemically recyclable polymer systems cannot be considered mature for commercial applications. With the motivation of eliminating plastic waste in the environment, alongside limiting energy and material losses, it is certain that new commercially attractive polymer systems will arise. This will require both expertise and close cooperation between polymer and organic chemists. It is thereby predicted that the coming decades will be a theater of significant innovation regarding the exploration, and ultimately widespread application, of chemically recyclable polymers.
